# Shape-Controlled Synthesis of Luminescent Hemoglobin Capped Hollow Porous Platinum Nanoclusters and their Application to Catalytic Oxygen Reduction and Cancer Imaging

**DOI:** 10.1038/s41598-018-32918-w

**Published:** 2018-09-28

**Authors:** Fatemeh Molaabasi, Morteza Sarparast, Mojtaba Shamsipur, Leila Irannejad, Ali Akbar Moosavi-Movahedi, Abouzar Ravandi, Behnam Hajipour Verdom, Reza Ghazfar

**Affiliations:** 1 Department of Biomaterials and Tissue Engineering, Breast Cancer Research Center, Motamed Cancer Institute, ACECR, Tehran, Iran; 2 Department of Chemistry, Faculty of Basic Sciences, Tarbiat Modares University, Tehran, 14115-175 Iran; 3 Department of Chemistry, Michigan State University, East Lansing, Michigan, 48824-1322 United States; 4 Department of Chemistry, Faculty of Basic Sciences, Razi University, Kermanshah, Iran; 5 Institute of Biochemistry and Biophysical Chemistry University, Tehran University, Tehran, Iran; 6 Department of Chemistry, Faculty of Basic Sciences, Sharif University of Technology, Tehran, Iran; 7 Department of Biophysics, Faculty of Biological Sciences, Tarbiat Modares University, Tehran, 14115-154 Iran

## Abstract

Engineering hollow and porous platinum nanostructures using biomolecular templates is currently a significant focus for the enhancement of their facet-dependent optical, electronic, and electrocatalytic properties. However, remains a formidable challenge due to lack of appropriate biomolecules to have a structure-function relationship with nanocrystal facet development. Herein, human hemoglobin found to have facet-binding abilities that can control the morphology and optical properties of the platinum nanoclusters (Pt NCs) by regulation of the growth kinetics in alkaline media. Observations revealed the growth of unusual polyhedra by shape-directed nanocluster attachment along a certain orientation accompanied by Ostwald ripening and, in turn, yield well-dispersed hollow single-crystal nanotetrahedrons, which can easily self-aggregated and crystallized into porous and polycrystalline microspheres. The spontaneous, biobased organization of Pt NCs allow the intrinsic aggregation-induced emission (AIE) features in terms of the platinophilic interactions between Pt(II)-Hb complexes on the Pt(0) cores, thereby controlling the degree of aggregation and the luminescent intensity of Pt(0)@Pt(II)−Hb core−shell NCs. The Hb-Pt NCs exhibited high-performance electrocatalytic oxygen reduction providing a fundamental basis for outstanding catalytic enhancement of Hb-Pt catalysts based on morphology dependent and active site concentration for the four-electron reduction of oxygen. The as-prepared Hb-Pt NCs also exhibited high potential to use in cellular labeling and imaging thanks to the excellent photostability, chemical stability, and low cytotoxicity.

## Introduction

In recent years, application of Platinum as an excellent active catalyst has raised an interest in catalysis including oxidizing carbon monoxide^1^, reducing oxygen^2^, and oxidatively dehydrogenating propane^3^. However, its commercial applications as an electrocatalyst are restricted because of high price and scarce resources. Oxygen reduction reaction (ORR) is of great importance in electrochemical energy conversion and storage devices for which many efforts have been devoted for developing a new synthetic strategy to obtain Pt-based nanomaterials with different compositions and structures to achieve enhanced performances^4^. For example, Adzic’s group demonstrated that Pt hollow nanocrystals can be used as a suitable catalyst in ORR, since they exhibited sustainable enhancement simultaneously in durability and Pt mass activity in acid fuel cells^5^. Additionally, Chunyu Du’s group reported that the activity and stability of Pt nanocrystals toward ORR can be attributed to the synergistic contribution of the optimized Pt facets, whereas the Pt octahedrons with (111) facets exhibit higher specific activity compared to Pt cubes with (100) facets in HClO_4_ solutions^6^.

Controlling the morphology of Pt nanocrystals during the chemical reduction strategy was provided by applying different capping agents such as macromolecules or polymers, negatively or positively charged ions, carbonyl compounds, and trace metals; well-nigh all of them are non-eco-friendly and have suffered from complexity, high cost, and blocking active sites of Pt surfaces by capping agents like alkanethiols in micellar surfactants^6^. Thus, there is a strong demand towards the synthesis of Pt nanocrystals that are well-controlled surface and morphology (including size and shape) via a simple, versatile and environment-friendly method.

As the catalytic activity of platinum nanoclusters (Pt NCs) is mostly influenced by (i) cluster size and/or the number of atoms in cluster^7^, (ii) cluster shape^8^, and (iii) cluster coverage^9^; an ideal supporting or template material become a great demand in synthesizing and size/shape control of Pt nanoclusters. In spite of significant progress in controlling the size of Pt NCs via the gas-phase deposition, a few successes were obtained via chemical reduction methods^7,10^; Besides, to date no report has yet been found for controlled shapes of fluorescent metal nanoclusters, and likewise there are few reports and applications for fluorescent Pt NCs so that the presented protocols for synthesis of fluorescent Pt NCs usually require multiple steps and toxic organic solvents or additive agents^11,12^. For example, Wang’s group synthesized poor stable Pt_20_ NCs using BSA in the presence of NaBH_4_ as a toxic reducing agent^13^. In another report, yellow-emitting Pt NCs (Pt@GSH) have been synthesized during two steps with the ligand etching process^14^; However, they did not exhibit electrocatalytic properties such as ORR activity. This can be due to the presence of protecting ligand on the active surface of clusters limiting their electrocatalytic applications^15^.

To date, biomimetic synthesis of noble metal nanostructures with programmable control of crystal growth is an attention approach, thereby creating desirable size, shape, and functions^16^. Considering the roles of biomolecules on mineralization, this is achieved by means of self-assembling of protein cages with intrinsic nanometer dimension of the cage inner cavity and specificities such as heat shock protein^17^, engineered P22 coat protein^18^, ferritins^19^, and ferritin-like proteins^20^. peptides of specific sequences also can selectively bind to a particular crystal facet and lower the order of surface energy; for example, Huang’s group has reported the use of two facet-specific peptides that selectively stabilize the {100} and {111} faces of platinum, yielding platinum nanocubes and nanotetrahedrons, respectively^21^. Others have also suggested that surface modification of the inner cavity of the ferritin cage by do-decapeptide -Asn-Pro-Ser-Ser-Leu-Phe-Arg-Tyr-Leu-Pro-Ser-Asp-(AG4) caused to enhance the binding affinity for the growth of silver nanoparticles^22^. However, there is relatively little insight into structure-property relationships for the controlled synthesis of fluorescent nanocrystals using peptides and proteins as biotemplates mediating particular growth directions.

Bearing these important issues in mind, in this study, the most important discovery is dealing with the shape-controlled synthesis of fluorescent Pt NCs with a strong shell effect of Hb, accompanied by aggregation-induced emission (AIE) effect due to oriented attachment and Ostwald ripening mechanisms; the interesting results which are totally different from the commonly used proteins, i.e., Lys and BSA, as stabilizing agents for the synthesis of Pt NCs^13,23^. In fact, we have used a facile, one-pot and green strategy, i.e. direct-reduction approach, for the preparation of fluorescent platinum nanoclusters in hemoglobin (Hb) caped by controlling the following parameters: temperature, Pt:Hb molar ratio, and incubation time. The proposed method does not require hazardous reducer (such as NaBH_4_), surfactant, and the addition of a high amount of platinum precursor, presenting a novel and environmentally friendly method to bio fabricate regular hollow polyhedra Pt NCs.

We have found the potential application of Hb/Pt NCs in electrocatalytic oxygen reduction owing to the unique hollow porous structure of Pt crystals^24^. Therefore, their electrocatalytic criteria such as mass and specific activity, durability, and the number of transferred electrons have been investigated regarding the formation reaction time, morphology, surface structure, surface to volume ratio, and the content of aggregated platinum nanoclusters. Also, low toxicity, excellent photostability, and environmental stability over a wide pH range as well as easy functionalization of the outside Hb layer make the Hb/Pt NCs a promising potential for bioimaging and drug delivery. In this regard, we designed a bioimaging system, Pt@Hb/HA, containing two components: HA (hyaluronic acid) and Pt@Hb for Hela cells as cancer cell model in which HA specifically interact with transmembrane glycoprotein CD44 receptor, which overexpresses on surfaces of various cancer cells. Therefore, it is probably fair to claim that this research combines the advantages of fluorescence properties based on AIE effect and electrocatalytic activity of Pt NCs resulting in universal applications to both bioimaging and ORR activity. Additionally, based on the mechanistic information obtained from successful experiments, this study can provide useful insights into a biomimetic material design utilizing engineered proteins or peptides to predict synthesis of shape-controlled NCs exposing selective surfaces which will be applicable to catalysis, optoelectronics, and medical applications.

## Results and Discussion

### Formation of hemoglobin protected Pt clusters

**Figure 1:**
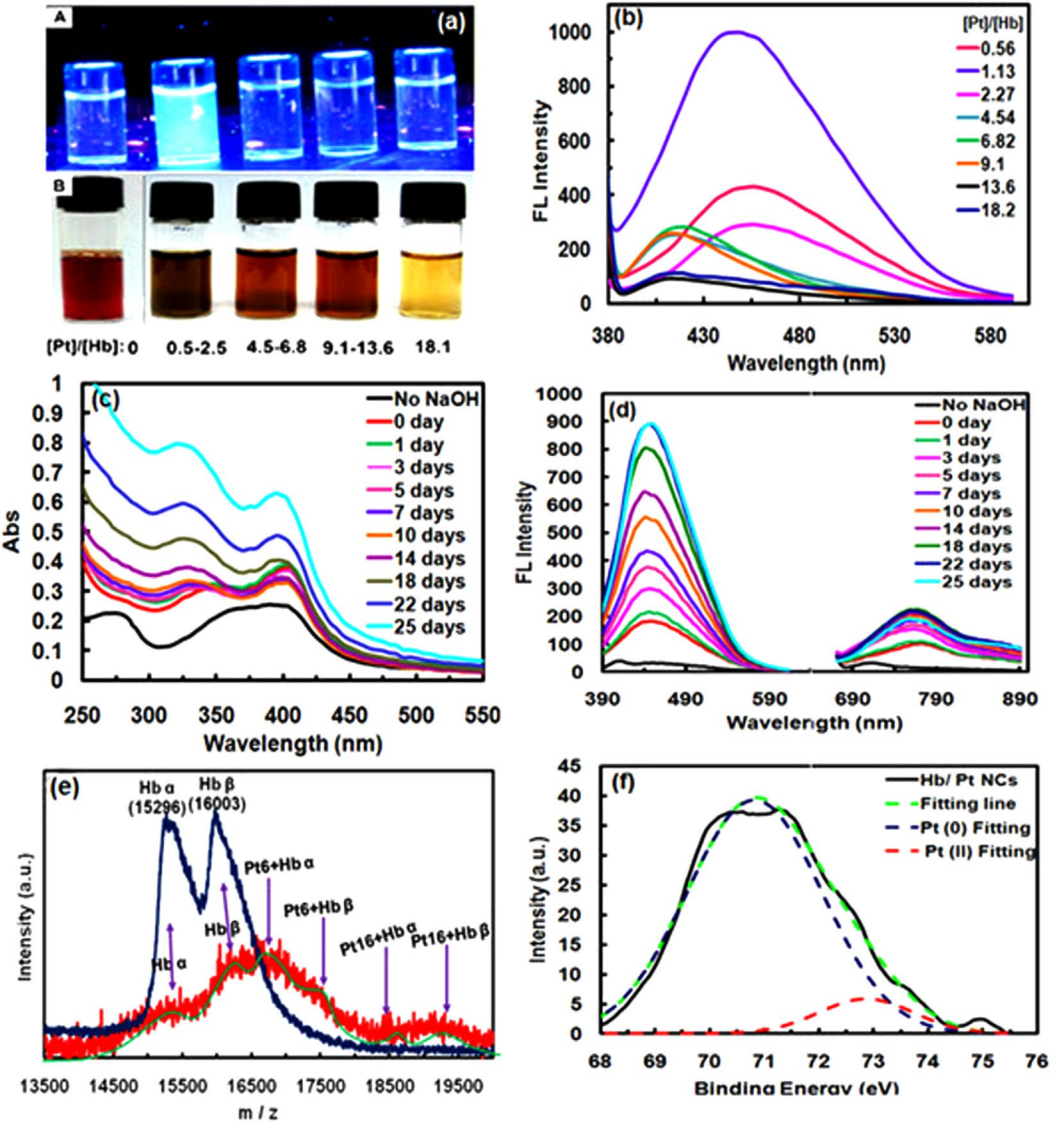
Photophysical characterization and optimization. (**a**) Photographs of the Hb/Pt NCs with different molar ratios of Pt/Hb under (A) UV illumination (λ_ex_ = 365 nm) and (B) visible light. (**b**) Emission of as-prepared Pt nanoclusters with different molar ratios of Pt/Hb at the same Hb concentration (0.11 mM). (**c**) UV–vis and (**d**) Emission spectra of Hb/Pt NCs obtained from monitoring reaction times with 0.11 mM Hb and 0.125 mM H_2_PtCl_6_ at 37 °C. (**e**) MALDI MS data of Hb (blue) and Hb/Pt NCs (red) collected after 22 days. Shift in mass was compared against the mass of Hb at pH ∼ 12.4. (**f**) X-ray photoelectron spectrum of Pt 4f_7/2_ for the Hb/Pt NCs obtained after 22 days at 37 °C.

Hemoglobin (Hb) was used as both reducing and capping agent for direct reduction of Pt^4+^ ions resulting in synthesis of luminescent Pt NCs under one-step “green” process; typically, Pt NCs are prepared by mixing H_2_PtCl_6_ and Hb solution at 37 °C for 10 min, followed by NaOH addition (pH ~ 12). The photographs, fluorescence, and UV-vis spectra of Hb/Pt NCs with different molar ratios of Pt: Hb during 5 days revealed 1.14 as optimized ratio (Fig. 1a,b; Supplementary Fig. S1). For the Pt/Hb ratio of 1.14, Hb/Pt NCs showed the most intense fluorescence at λ_em_ = 450 nm in alkaline reaction media (pH ~ 12.4), but not reached the equilibration after 5 days (See Supplementary Fig. S1). Thus, the time evolution of UV-vis (Fig. 1c) and fluorescence spectra for clusters were investigated from 1 h to 25 days, and it appeared that the reaction was almost completed in about 22 days (Fig. 1d). Moreover, compared to Au NCs, prepared under the same conditions^25^, Hb/Pt NCs exhibited near-infrared (NIR) fluorescence emission at λ_em_ = 760 nm (λ_ex_ = 320 nm) attributed to a larger cluster size^14,26^. The emission intensity at 760 and 450 nm reaches a maximum after about 22 days (Fig. 1d). Thus, the solution contains two species, a blue emitting cluster, and a red emitting Hb/Pt NCs system, when Pt NCs were synthesized with a low concentration of H_2_PtCl_6_ (0.125 mM). Since the PL band at 760 nm probably arises from the surface state^26–29^, the emission at 450 nm is much higher than that of 760 nm. This finding could be due to a low fraction of Pt (II) species on the cluster surface (~13.68%) as discussed below in XPS spectra, which is similar to the emission spectra of Ag NCs^26^ and in contrast to the emission spectra of Cu NCs in the previous reports^29,30^. The large Stoke’s shift (440 nm) suggests that the NIR (760 nm) emission from aggregated Pt(II)–X complexes was mainly phosphorescence and could be attributed to metal-centered triplet excited state considering the ligand-to-metal charge transfer (LMCT) or LMMCT behavior from Pt(II)-Hb complexes, i.e. from oxygen and nitrogen atoms in the amino acids to the Pt(II) ions), to the Pt atoms and subsequent radiative relaxation, which can be enhanced by Pt–Pt interactions^26,28–31^. Detailed synthetic procedures as well as investigation of the effective kinetic parameters such as temperature and external reducing agent (NaBH_4_) are elaborated in Supplementary Figs 2–4.

The clusters composition as a significant factor in determining the chemical and physical properties of these nanoplatforms was elucidated by matrix-assisted laser desorption ionization-time of flight (MALDI-TOF) experiments. As shown in Fig. 1e there are two different clusters, Pt_6_ NCs and Pt_16_ NCs, which are consistent with the two fluorescence emissions as discussed above. In fact, it can be concluded that the blue emission is related to Pt_6_ NCs and the weaker red emission is related to Pt_16_ NCs. (Supplementary Page S6 for more information). It should be noted that the reduced mass spectral intensity with a substantial broadening of the features in Hb/Pt NCs compared to free Hb (Fig. 1e), the fact that Pt NCs may be able to alter protein structure leading to better cluster formation^26,32,33^. The successful synthesis of Pt nanoclusters was also confirmed by X-ray photoelectron spectroscopy (XPS) as shown in Fig. 1f. The Pt NCs revealed a characteristic peak of zero-valence Pt at 70.8 eV assigned to 4f_7/2_, indicating the reduction of Pt (IV) during the formation of the cluster. After deconvolution of 4f_7/2_ binding energy, two distinct components were found the more intense Pt (0) centered at 71.0 eV, and the weaker intense Pt (II) centered at 72.8 eV that may correspond to the charge transfer in the Pt-S bonds^14,34^. Furthermore, the quenching of solution with addition of Hg^+2^, which can bind to Pt(0) (4f^14^5d^10^) through metallophilic interactions (Supplementary Fig. S5a), and of cysteine, which can interact with Pt (II) ions via the formation of Pt(II)-thiolate complexes and causes to removal of Pt (II) on the surface (Supplementary Fig. S5b), could also confirm the presence of Pt (0) and Pt(II) in a core-shell structure model (or Pt(0)@Pt(II)-Hb NCs)^27,35,36^.

**Figure 2:**
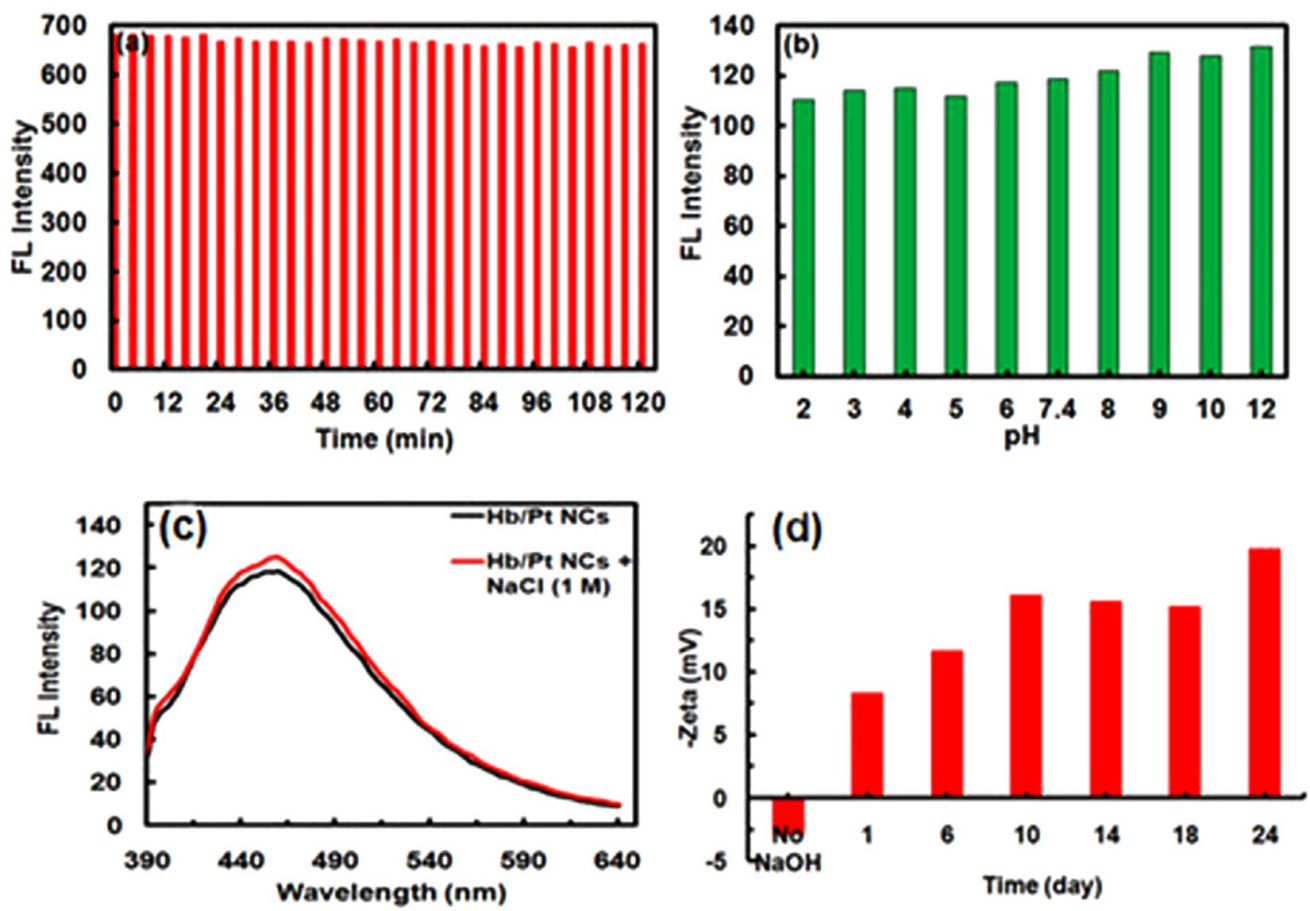
Stability tests. (**a**) Photostability of Hb/Pt NCs after ultraviolet (UV) irradiation for up to 2 hours with λ_ex_/λ_em_ = (320 nm)/(450 nm). (**b**) The pH effect on the fluorescence emission intensity of Hb/Pt NCs; [PBS] = 10 mM. (**c**) Emission spectra of Hb/Pt NCs in the absence and presence of 1 M NaCl. (**d**) Zeta potential of Hb/PtNCs monitored with time.

The stability test of Hb/Pt NCs was investigated in different experimental conditions including pH, ionic strength, and UV radiation, by monitoring their fluorescence properties (Fig. 2). No obvious change in fluorescence intensity was observed for Hb-Pt NCs in solutions after ultraviolet (UV) irradiation for up to 2 h (Fig. 2a). The great UV stability of Pt NCs may be originated from the large size of hemoglobin used as template (574 aa) and, consequently, the effective coverage of surface atoms^26^. The stability of as-prepared Pt NCs was also evaluated as a function of pH, in which no significant change has been observed, demonstrating the high stability of the as-prepared nanoclusters at different pHs (Fig. 2b). It should be noted that an approximately 10–15% higher fluorescent intensity at λ_em,max_ with no shift in maximum wavelength was observed under basic condition (at pH > 7.4). This is because of the fact that, at higher pHs, the hemoglobin cage (with a pI of 6.8) on the surface of the Hb/Pt NCs is deprotonated, which weakens the affinity of aggregation and, thereby, the stabilizing system^37^.

Furthermore, the Hb/Pt NCs exhibited high stability in s solution of high salt concentration (e.g., 1 M NaCl) (Fig. 2c). The stability of the Pt NCs was also examined by measuring the zeta potential as a function of incubation time (Fig. 2d). The zeta potential of the Pt NCs was increased from −8.3 (1 day) to −16.2 ± 2 mV (10 days) and reached to ~−20 ± 2 mV at 24 days, reflecting a good electrostatic repulsion between the clusters^25^. This was further supported by TEM images with highly-dispersed Pt particles in the following section^26^. Thus, from the stability tests it could be concluded that the as-prepared Hb/Pt NCs are highly stable in different pH ranges, at high salt concentration (e.g., 1 M NaCl), and after ultraviolet (UV) irradiation for up to 2 h. Moreover, the powder of the Hb/Pt NCs was stable for at least 3 months at room temperature and ~12 months at 4 °C, so that the fluorescence intensity of the as-prepared Pt NCs remain about 75–90% of their initial state^26^.

### Shape-controlled synthesis of Hb/Pt NCs upon aggregation-induced emission

To get insight into the PL mechanism, the corresponding UV-Vis spectra of pure Hb and Hb/Pt NCs solutions at 37 °C were studied more precisely with time from 0 day to 22 days (Fig. 1c). As time progressed, the absorption band of as-prepared Hb/Pt NCs at 350 nm gradually increased because of enhancing of the background scattering due to an increase in the aggregation degree of nanoparticles with disparate core size (Fig. 1c)^38^. Furthermore, the broad absorption peak of Hb/Pt NCs at below ~600 nm gradually increases over time indicative of scattering contribution upon the formation of the larger size of the Pt NCs aggregates. The absorption band at 350 nm (0 day) was also shifted to 320 nm (14 days) over time; however, no obvious shift was seen in maximum emission at 450 nm indicating that the size of the cluster (number of atoms for one molecule) was almost retained^25^ (Fig. 1d). Besides, the characteristic peak of Hb at 280 nm (resulted from the phenyl group of Trp and tyrosine residues) was distorted or hidden with the incorporation of platinum nanoclusters into protein. Additionally, the absorption band at ~410 nm is blue-shifted to 395 nm and weakened (Supplementary Fig. S6). The phenomena indicating that *in situ* formation of Pt NCs aggregates distorts the environment surrounding of heme groups (Supplementary Fig. S6).

**Figure 3:**
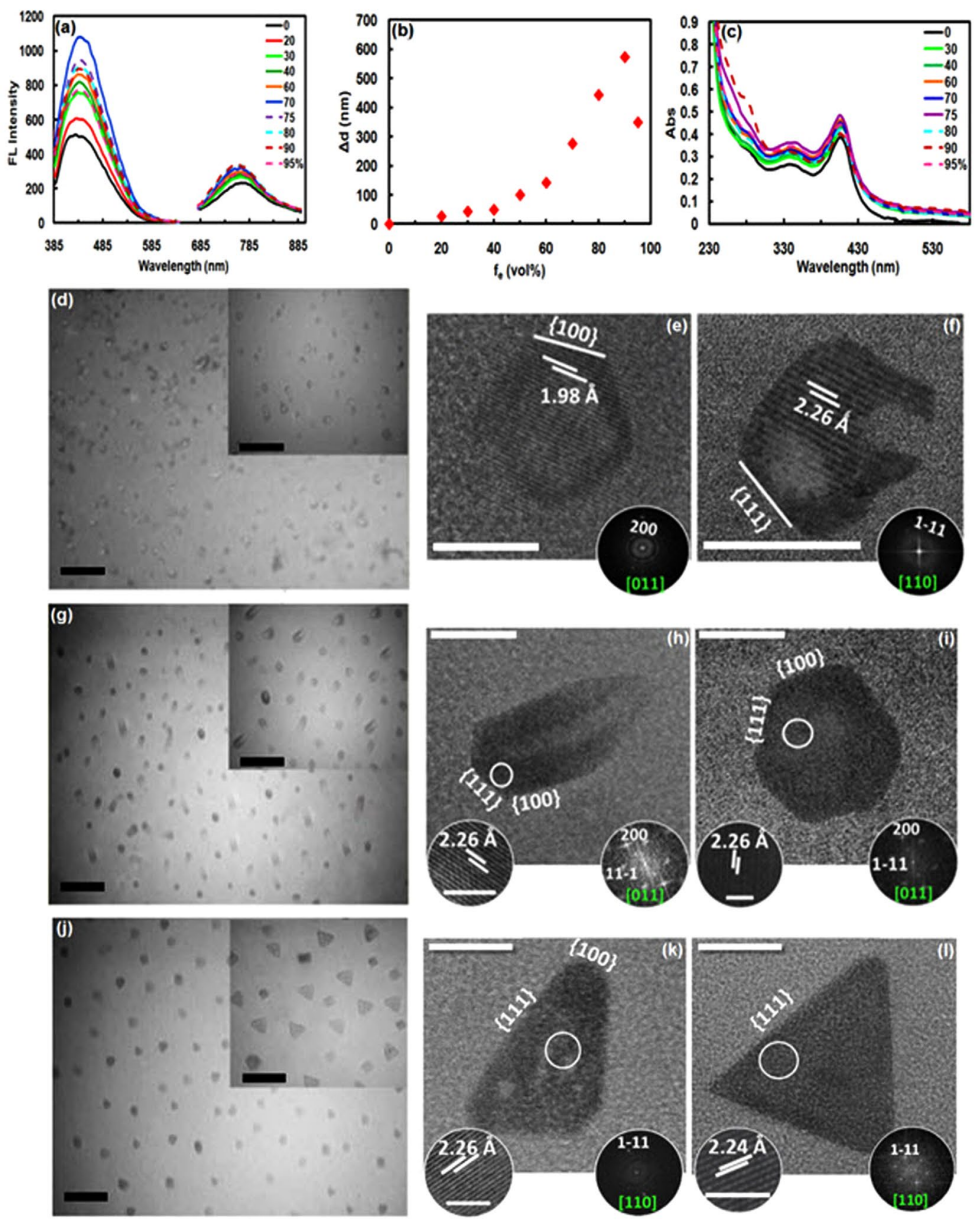
AIEE phenomenon and shape evolution of Hb-regulated porous hollow luminescent platinum tetrahedrons. (**a**) Emission spectra of Hb/Pt NCs in mixed solvents with different *f*_*e*_ at λ_ex_ = (320 nm)/(450 nm) and λ_ex_/λ_em_ = (320 nm)/(760 nm). (**b**) Size distributions and (**c**) UV−vis spectra of Hb/Pt NCs versus *f*_*e*_. The results were recorded at 30 min after mixing Hb/PtNC solution (1.1 µM) with different ethanol concentrations. TEM and HRTEM images of the porous hollow Pt nanocrystals obtained from mixtures of 0.11 mM Hb and 0.125 mM H_2_PtCl_6_ in the over reaction time at 37 °C: 1 day (**d**–**f**), 10 days (**g**–**i**), and 22 days (**j**–**l**). Insets are high resolution images of the marked areas (left) with the corresponding FFT patterns (right). Scale bars: 100 nm for TEM images and 50 nm for corresponding insets shown in (**d**,**g**,**j**); 5 nm for (**e**,**f**); 10 nm for (**h**,**i**); 20 nm for (**k**,**l**); 2 nm for inset circles shown in (**h**,**i**,**k**,**l**).

These findings imply the aggregation-induced emission enhancement (AIEE) phenomenon in Pt NCs capped by hemoglobin that recently has observed in nanocluster platforms such as cysteamine (CSH)/Au NCs^39^, GSH/Au NCs^28^ and ascorbic acid (AA)/Cu NCs^30^. To get a clear insight into the AIEE effect, solvent-induced aggregation approach was employed on the PL behavior of the Pt NCs by using ethanol/water mixtures in which the polarity of the mixed solvent was controlled with different concentration of ethanol to obtain a set of fractions (*f*_*e*_, vol%). As illustrated in Fig. 3a, the PL intensity at 450 and 760 nm progressively intensified with increasing of *f*_*e*_ from 20% to 70%, likely due to inter-aggregation of Pt NCs that makes larger aggregates^26,39^; after that the PL intensity at both wavelengths gradually dropped with increasing of *f*_*e*_ and an obviously decrease was happened at *f*_*e*_ 95%, in which the observable precipitates were appeared^28,30^. This behavior was also supported by the size measurements with DLS analysis (Fig. 3b)^28,30^. Furthermore, the background scattering or absorbance tails was intensified in the UV region by increasing in *f*_e_ and then decreased for *f*_e_ > 75%, a nearly-finding corresponding to DLS and PL observation, demonstrating large nanocrystal formation^26,28,38^ (Fig. 3c). This phenomenon clarifies AIEE characteristics of Pt NCs in weakly polar solvents which disrupted the hydration shell of Pt(II)–X complexes on the surface of Pt core, thus charge neutralization and consequently, complex aggregation happened. This facilitated platinophilic (Pt···Pt) interactions and dense aggregates leading to an enhance in the restricted intramolecular motions in the aggregates^26,30^. In fact, the similarity between the evolution of Pt(0)@Pt(II)-Hb NC aggregates in the alkaline solution and aggregated complexes in ethanol/water mixture, in terms of spectral features, suggests that the emission of Pt NCs was derived from the AIEE effect which has had a significant impact in various field-effect biocatalysts and bioaffinity based biosensors.

To further investigate the effect of AIEE, the as-synthesized Pt NCs were analyzed by transmission electron microscopy (TEM) coupled with high resolution (HR) and Fourier transform (FT) patterns, by which a surprisingly shape-controlled Pt nanoclusters enclosed by particular facets was clearly observed under AIEE effect^40^. In this way, the time-dependent experiments were conducted and the morphology evolution (i.e. size and shape) of Pt NCs aggregates were studied at different reaction times of 1^st^ day, 10^th^ day and 22^nd^ day (Fig. 3d–l). On the 1^st^ day of reaction, the Pt NCs aggregates showed hollow spherical-like particles with an average diameter of 8.7 ± 1.3 nm (yield of 90%) (Fig. 3d) and a wall thickness of ~2.4 nm. The HRTEM image in Fig. 3e,f shows typical irregular polyhedral nanocrystals enclosed by {100} and {111} facets, as confirmed by the Fourier transform (FT) pattern along the [011] zone axis (Fig. 3e,f). The obvious contrast of dark edge and light center in the images indicates that hollowing takes place in the interior of Pt NC aggregates, followed by a solid core filling hollow aggregates with the time evolution from 1^st^ to 10^th^ day, as evidenced in Fig. 3g; Actually, with an increase in reaction time to 10 days, the size of resulting hollow particles increased to 18.0 ± 1.8 nm with a typical thickness of 3.7 ± 0.7 nm, having approximately unusual polyhedra with outward trail void, nonuniform shell thicknesses, and asymmetrical NP shapes, confirming that particle growth and nucleation occurred in parallel during the shape evolution process (Fig. 3g,h). Additionally, there are hollow hexagonal like nanoaggregates having a typical size of 10.4 + 0.8 nm and a wall thickness of 3.5 ± 0.2 nm that can be recognized as truncated octahedrons bound by {100} and {111} facets, corresponding to the HRTEM image and FT pattern along the [011] zone axis (Fig. 3i). The presence of relatively flat areas separated by edges implies that the cluster aggregates are not spherical (Fig. 3i). Furthermore, according to the TEM images, it is found that the Pt truncated octahedron-like Pt nanocrystals having smaller truncated facets can also be connected to another truncated octahedron resulting the formation of larger unusual truncated octahedra (Fig. 3g). It seems that hemoglobin molecules selectively can bind to Pt-{111} facets and lowered their surface energy, thereby stimulating more growth along the {100} directions with evolution time from 1 to 10 days. After the complete reduction of the Pt sources (22^nd^ day), the hollow nanocrystals evolved into tetrahedrons with hollow interiors and an average diameter of 19.7 ± 2.2 nm, some of which were truncated tetrahedrons (Fig. 3j–l) with much more growth in the <100> than in the <111> direction compared to unusual truncated octahedron-like shapes (Fig. 3h); it should be noted that after 22 days unusual hollow polyhedral or/and unusual truncated octahedron was decreased (~1%) and small number of porous octahedrons, and cubes or slightly truncated octahedron were observed (not shown). The mean shell thickness of 3.6 ± 0.26 nm remains approximately constant (Fig. 3k,l) similar to the thickness of polyhedral shapes obtained on 10^th^ day (Fig. 3h,i); this may be due to rearrangement of atoms on the surface of the nanocrystal induced by interaction between the binding polypeptide chains of Hb and the surface atoms of clusters, leading to the formation of nanocrystal shapes with the low surface energy of {111} facets^21^. Lattice analysis by HRTEM further revealed the single crystal nature of resulting tetrahedral Pt NCs enclosed by four {111} facets (Fig. 3l). In addition to triangular shaped structures, the diamond-like shape of nanocrystals (Fig. 3j) further confirming that the predominant shape of Pt nanoclusters is tetrahedron when hemoglobin was used as capping agent in alkaline media at 37 °C^21^. The truncated tetrahedrons display {100} and {111} facets but predominantly enclosed by {111} facets as confirmed by the FT pattern along the [110] zone axis (Fig. 3k).

We found overall that longer reaction time leads to the formation of larger platinum nanocrystals with higher concentrations. This high aggregation tendency of Pt nanoclusters can be due to their high surface energy. From these observations, it can be concluded that the nanocrystals with hollow interiors mainly evolved from {111} and {100} enclosed hollow truncated octahedrons as well as unusual hollow polyhedra with outward trail void, known as probably unusual truncated octahedron to truncated tetrahedron, and eventually {111}-only enclosed tetrahedrons in which hemoglobin act as the active biomolecule for Pt nanocrystal formation. It is expected that Hb α- and β-chains can selectively bind to stabilize Pt-{111} facets, lowering the order of specific surface free energy of Pt-{111}, and increasing growth rate along the <100> directions, thereby gradually increasing the ratio of {111} facets to {100} facets, and consequently leading to the formation of well-defined tetrahedral nanocrystals enclosed by {111} facets only^21,41^. To further demonstrate the importance of surface-specific hemoglobin molecules in controlling and regulating of shape and size of aggregated nanoclusters, the thermodynamic control needs to be considered. Thus, a control experiment was carried out using BSA as capping agent in order to compare the ability of other proteins to promote the formation and/or to control the shape of Pt nanocrystals in the same typical experimental conditions^21,41^. The usage of BSA resulted in Pt nanoclusters with an average diameter of 2.7 nm which distinctively different from tetrahedrons (Supplementary Fig. S7), suggesting different platinum-binding patterns on albumin^13,23^, and confirming the effect of protein conformation on protein reactivity with the Pt ions and the pivotal role of facet-specific hemoglobin chains in thermodynamic of shape-controlled synthesis of platinum nanocrystals^21,41^. However, further investigation will be required to recognize the surface-specific amino acid sequence of the α- and β-chains, which preferentially bind to the Pt {111} surface. For this reason, a computational approach based on density functional theory (DFT) is under investigation to understand this subject which can be utilized for the biomimetic synthesis of shape-controlled nanocrystals in the near future^21,41^. It should be noted that some one-dimensional nanoribbons also have been seen which need more study to fully characterize and finding the mechanism of them and is under investigation.

**Figure 4:**
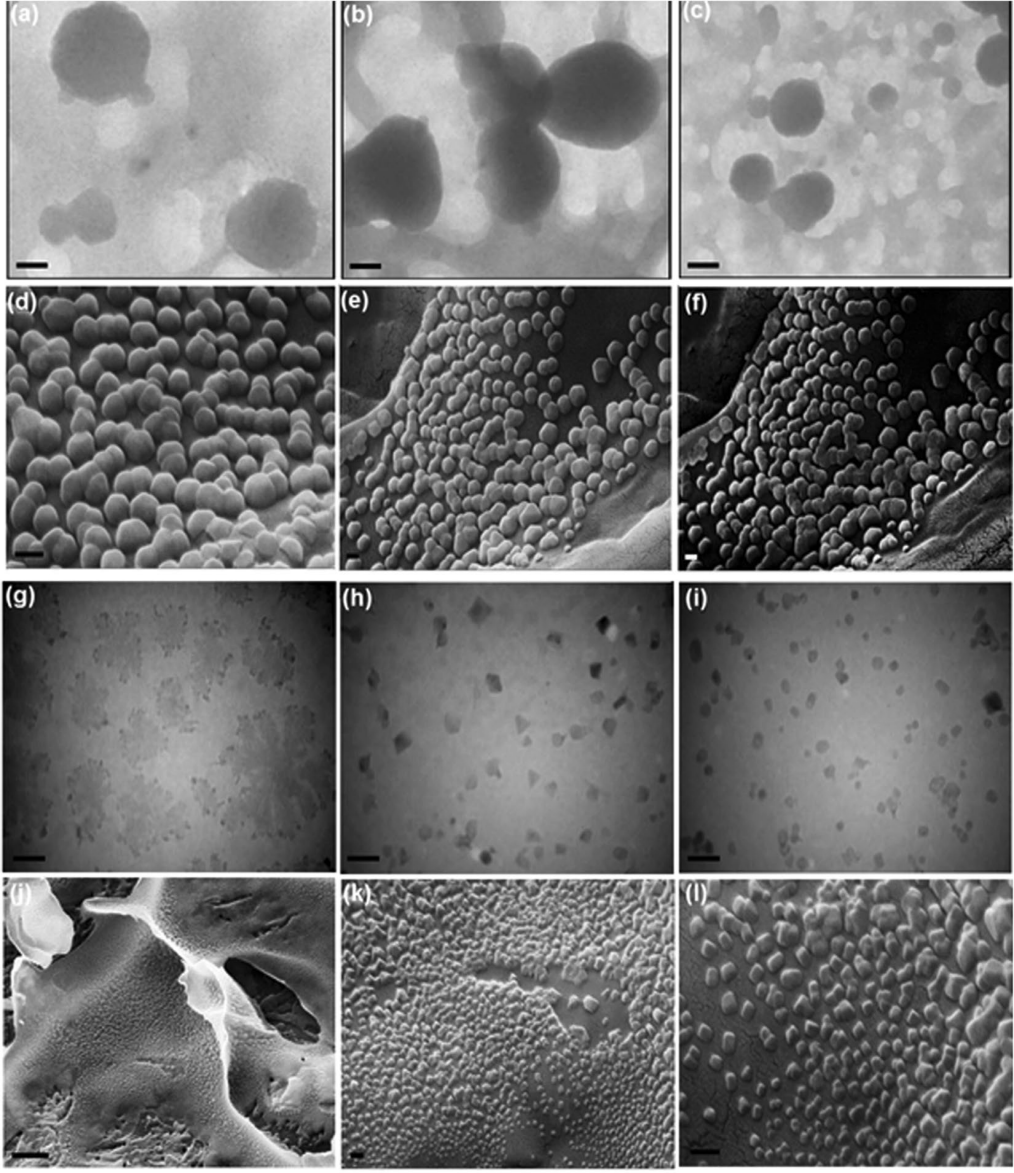
Kinetically-controlled growth of Pt NCs. TEM and FESEM images of the PtNC porous microspheres (**a**–**f**) and PtNC hollow polyhedra (**g**–**l**) obtained by mixing 0.125 mM H_2_PtCl_6_ in a 0.11 mM Hb solution at 67 °C in the absence of NaBH_4_ and at 37 °C in the presence of NaBH_4_, respectively. (**f**) Corresponding dark-field FESEM image of the PtNC porous microspheres shown in (e). Scale bars: 40 nm for (**a**); 80 nm for (**b**); 150 nm for (**c**); 100 nm for (**g**); 60 nm for (**h**); 35 nm for (**i**); 2 µm for (**j**) and 200 nm for (**d**–**f**,**k**,**l**).

Interestingly, Pt cluster seeds could direct evolve into irregular hollow polyhedra, unusual truncated octahedrons, truncated tetrahedrons, and then tetrahedrons via a kinetically induced asymmetric growth pattern (Fig. 3d–l). During the early stage of Pt NCs synthesis, the newly formed Pt atoms from the reduction of Pt precursor (PtCl_6_^2−^) initiate nucleation and subsequently grew upon clusters aggregation to form irregular polyhedral and then truncated octahedron enclosed by {100} and {111} faces with gradually increasing in sizes. In the later stage, in which Pt precursor was gradually consumed, the newly formed cluster (or nucleus) of Pt atoms, resulting from the reduction of H_2_PtCl_6_, tended to be deposited on the {100} facets of Pt truncated octahedron, and/or aggregated along the <100> direction during growth. In fact, the {100} has higher surface free energy compared to {111}, therefore, four of the eight {111} facets could be nucleated and grown, which leads to a morphology transformation from truncated octahedrons (Fig. 3i) to truncated tetrahedrons (Fig. 3k), and finally tetrahedrons (Fig. 3l)^41^. To further demonstrate the kinetically-controlled growth of Pt NCs occurring by AIEE process, the temperature was shifted from 37 °C to 67 °C and TEM/SEM images of the as-synthesized Pt NCs at 67 °C after 10 days were recorded while the mole ratio of Hb to Pt was still kept at 1:1.14^41,42^. The as-prepared Pt NCs displayed a uniform spherical-like morphology with high yield (Fig. 4a–f). This indicates that higher temperatures cause a faster reaction of the metal precursor and as a result of more compact crystallite aggregates produce. Thus, the crystal morphology switches from small hollow tetrahedrons obtained at 37 °C to large porous poly-crystalline microspheres at 67 °C (PPMs), accompanied by a drastic size change from 19.7 to 150 nm (Fig. 4a–c). In fact, the nucleation and growth occur faster at higher temperature and therefore cannot exist preferred growth directions^43^, which causes the near-spherical polyhedral morphologies with less distinctive single crystalline structures. These results could be attributed to distinctively AIEE effect on the size and shape of Pt NCs in which aggregation of nanoclusters can be obviously enhanced at higher temperature, whereas the asymmetric growth pattern obtained and allowed the formation of tetrahedrons at 37 °C to be lost at 67 °C^40,41^. FE-SEM images of the same samples (Fig. 4d–f) also suggest a large number of nanospheres with good uniformity and smooth surfaces, indicating strong interaction Pt surfaces with hemoglobin chains which allow it to provide effective covering as-prepared Pt NCs prepared by simple, reproducible strategy. Using NaBH_4_ as a strong reducing agent in the synthesis of Pt NCs at 37 °C under typical condition resulted in the formation of aggregated hollow polyhedral Pt NCs including tetrahedrons, cubes, octahedrons, spheres, truncated tetrahedrons, as well as large dendritic hollow networks including irregular structures with hollow interiors (Fig. 4g–l). This difference in presence of NaBH_4_ is because NaBH_4_ produces a burst of nucleation in a short time and causes various shape production by increasing the reaction rate which leads to the loss of selective facet binding on Pt surfaces, demonstrating the distinctive specificities of hemoglobin. Furthermore, although the reduction rate of Pt precursor could also be controlled by changing the temperature, it seems that heme iron (Fe^2+^ or Fe^3+^) can be critical for the kinetical controlling morphology thorough slowing down the reduction reaction of the Pt compounds. In fact, it mediated diffusion rate of Pt atoms deposited on the surface of a cluster seed toward the lowest-energy side faces and edges, thereby increasing the growth along the 〈100〉 direction to form tetrahedrons for a long period of time. By the addition of NaBH_4,_ however, heme degradation and/or releasing of iron from Hb take place according to the UV-vis measurements (Fig. S4b), causing a faster reduction and less shape-selective Pt NC aggregates formation^34,44,45^. According to the above results, the sensitivity and selectivity toward morphology, shape, and size of the Pt NCs aggregates depend on the type of capping agent, reaction time, temperature, and reducing agent, all of which could be due to different adsorption rate of precursor on the Pt (111) and Pt (100) facets. Additionally, the configuration of protein template can be tuned under these self-ordering conditions owning to the strong correlation between these parameters and high conformational flexibility of Hb.

### Formation mechanism of the Pt NCs aggregates

The FTIR and CD techniques have been used to study the formation mechanism of the Pt NCs in the Hb aqueous solution (Supplementary Fig. S9), in which the strong binding between the surface of nanocrystals and amino acid residues of the protein causes protein skeleton unfolds, a favorable change for the formation of the clusters. In fact, hydroxyl (i.e., tryptophan, threonine, tyrosine, and serine), thiol (i.e., cysteine) and amine (i.e., histidine, lysine, and arginine) groups, as excellent nucleating agents, are probably responsible for the resulting Hb/Pt NCs nanocrystals formation^46^. Furthermore, the loss of α-helix stability due to hydrogen bonds breaking leads to an α-β transition, and consequently results in changing of the structure of the heme group, and perturbation of microenvironments around the deprotonated aromatic amino acid residue^25,26^.

**Figure 5:**
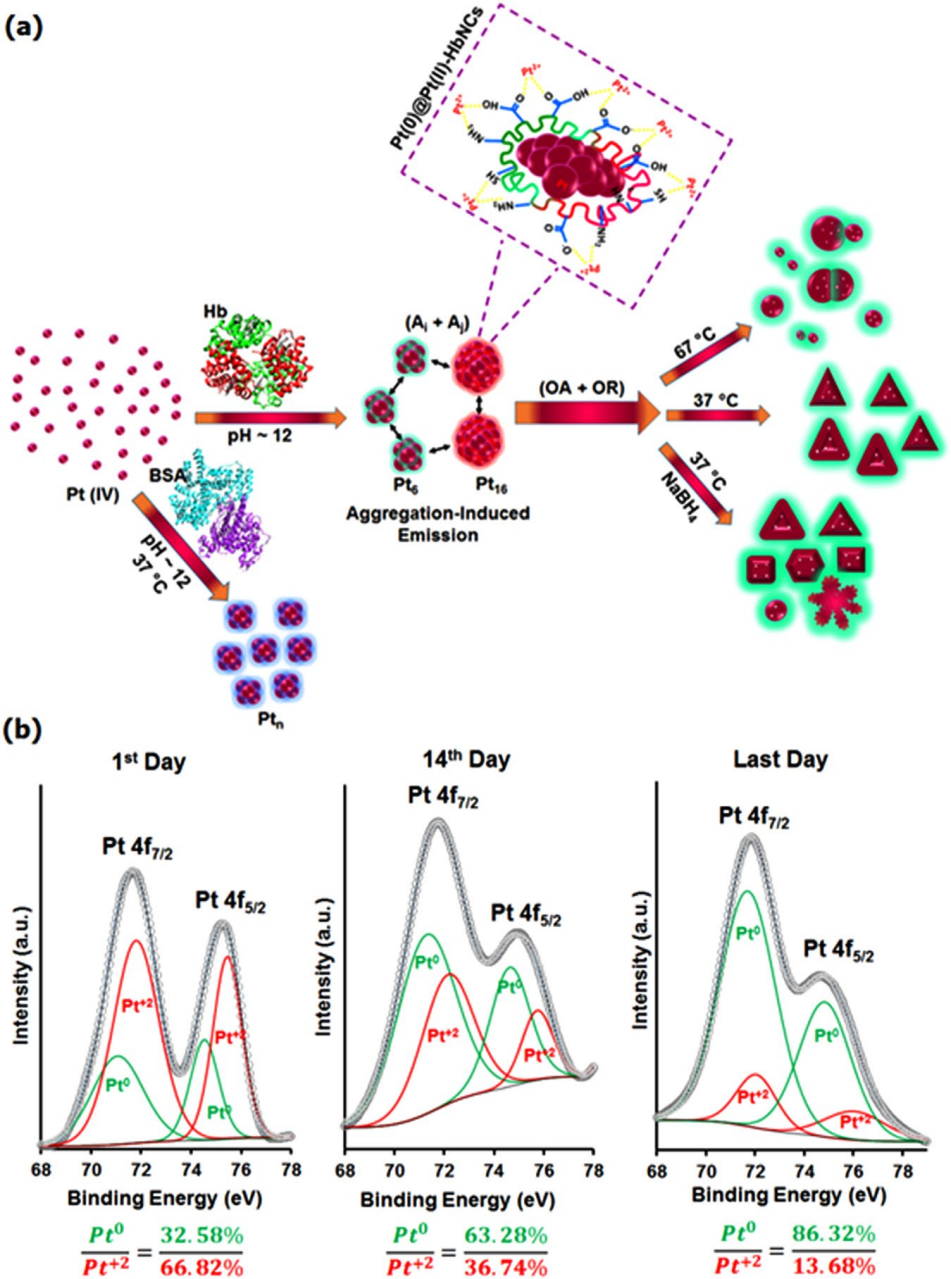
Formation mechanism of the Pt NCs aggregates. (**a**) Schematic illustration showing various porous hollow luminescent protein-templated platinum nanostructures synthesized via a combination of aggregation-induced emission, oriented attachment and Ostwald ripening mechanisms. (**b**) X-ray photoelectron spectrum of Pt 4 f for the Hb/Pt NCs obtained at three interval times of reaction (first day, middle day and last day) at 37 °C.

From the TEM complementary structural analysis of Hb/Pt NCs aggregates, the main formation mechanism for the Pt nanotetrahedrons has been understood well and are schematically shown in Fig. 5a. The present method seems to be consisted of four distinct periods. As depicted, there is a reasonable evidence that the shape- and size-controlled Pt NC hollow aggregates can be from an Ostwald ripening process assisted by oriented attachment in nanocrystal growth in two independent and competitive processes^47^. Overall, at the beginning stage of the formation of Pt NC aggregates, hydroxyl-, thiol-, and amine-groups in Hb can be coordinated with Pt (IV) ions and entrapped them, leading to the reduction of Pt (IV) to Pt (II)-X complexes (X: thiol, carboxyl, amine groups), followed by reduction of the oligomeric Pt (II)-X complexes to Pt (0) atoms via tyrosine/tryptophan residues under basic condition (~pH ∼ 12), and their subsequent sequestration by Pt(II)-X complexes. After a fast reduction of platinum ions, Pt_6_ and Pt_16_ clusters are actively formed, and small Pt nanoparticles are subsequently formed mainly by coalescence and aggregation of Pt(0)@Pt(II)−Hb NCs (nucleation-dominant formation period); additionally, the intra- and inter-complex platinophilic Pt(II)···Pt(II) interactions increase the degree of aggregation and/or the oligomerization of Pt(II)–X complexes, whereby the intramolecular vibrations and rotations of the complexes are restricted which blocks the non-radiative pathway and activates radiative decay, consequently, results in enhancing the luminescence^28–30,48,49^. This was further confirmed by XPS Pt 4 f spectra in which the intensity ratio of Pt(0) to Pt(II) peaks increased distinctly from 1 day to 25 days (Fig. 5b). In fact, the Hb molecules provide a compact shell on the surface of the Pt core surrounded by Pt(II)-X complex. Once the cluster nuclei (cluster seeds) were formed, and the growth of Pt nanoparticles on the seeds started via an oriented attachment process (OA), in which small crystallites attach each other through their suitable crystal facets according to their crystallographic directions, due to the differences in surface energy at each face (growth-dominant formation period)^40,47,50,51^. Simultaneously, solid evacuation takes place in the interiors of the Pt NC aggregates undergo Ostwald ripening process (OR) which results in the creation of hollow spherical-like particles. The driving force for the process is decreasing of total surface free energy according to the Gibbs-Thomson effect^50,52^. Therefore, the coarsening of Pt NCs is consistently controlled by the (OR + OA) mechanism^50,52^. The spaces generated in this process correspond to the regions where primary crystallites were either smaller or less dense in an aggregate and could be dissolved and re-deposited gradually from the central part toward the outer parts of the spherical-like aggregates. With time increasing, the size and shell thickness of hollow sphere-like increase, while the volume of voids slightly decrease, indicating OA as dominating stage can be attributed to the strong adsorption surface of capping agent on the nanoclusters. It is obvious that all the particles have an almost perfect oriented aggregation of nanoclusters in combination with surface recrystallization, leading to morphology change from sphere-like to unusual truncated octahedral with the outward trail void, i.e. the wall and the open-end region of Pt NCs, as a result of oriented assembly aggregation of primary nanocrystals. Then, the Hb molecules adsorbed on the perfect {111} surfaces of the truncated octahedrons can enhance the oriented assembly of these polyhedrons to form tetrahedral aggregates–oriented attachment of Pt NCs along <100> direction, and transform unusual hollow truncated octahedra to perfect hollow nanotetrahedrons. In this stage, it seems that the crystals migrated gradually outward due to Ostwald ripening which results in the expansion of interior space within the original aggregates and extension of recrystallization from the surface to the core, thereby further increasing the amount of void. In final, the crystal growth controlled by the OA and OR mechanisms produce porous hollow Pt tetrahedrons. It was noticed that during the OA growth, smaller particles of low mass diffuse faster than the larger particles of large mass, thus the faster growth rate for the smaller particles resulting in narrow size distribution^50,53,54^. Accordingly, the OA of nanoparticles plays a pivotal role in the growth process of monodisperse platinum nanocrystals formation and in controlling the size, shape, and crystallinity of nanocrystal aggregates; thus, the effect of OA growth factors such as type of capping agent, temperature, and reaction time on the synthesis of nanoclusters in the solution should be considered^52,54^ (see supplementary pages S11–14 for more details). It should be mentioned that the Kirkendall effect is based on the differences between the diffusion rates of two elements (DA ≠ DB), yielding a layer of shell materials (AB) upon thermal treatment. Because the hollow nanocrystals are synthesized without thermal treatment and also there is only a single phase (i.e., Pt NCs), the Kirkendall mechanism can be ruled out unambiguously^55,56^. Finally, as discussed in the next sections, the aggregation-based nanocluster growth leads to producing of clusters which not only have excellent ORR catalytic activity, due to their large surface area and stability, but also can be used in targeted cell imaging, thanks to their nontoxicity, ability to functionalization, and high luminescence quantum yield^40^, of which both are investigated in this report.

### Electrocatalytic performance of Pt NC aggregates

The ORR electrocatalytic activities of Pt NC aggregates were evaluated using a glassy carbon electrode modified with functionalized multi-walled carbon nanotubes and hemoglobin-capped platinum clusters with ultra-low Pt loadings that are: 0.68, 0.42, 0.38 μg_Pt_/cm^2^ disk for prepared Pt NCs at 67 °C, 37 °C, and 37 °C in the presence of NaBH_4_, respectively (Supplementary Fig. S9). CVs were measured in O_2_− or N_2_-saturated in 0.1 M HClO_4_ solution at a potential scan rate of 0.1 V s^−1^. All the currents were normalized to the geometric surface area of the GC electrode.

**Figure 6:**
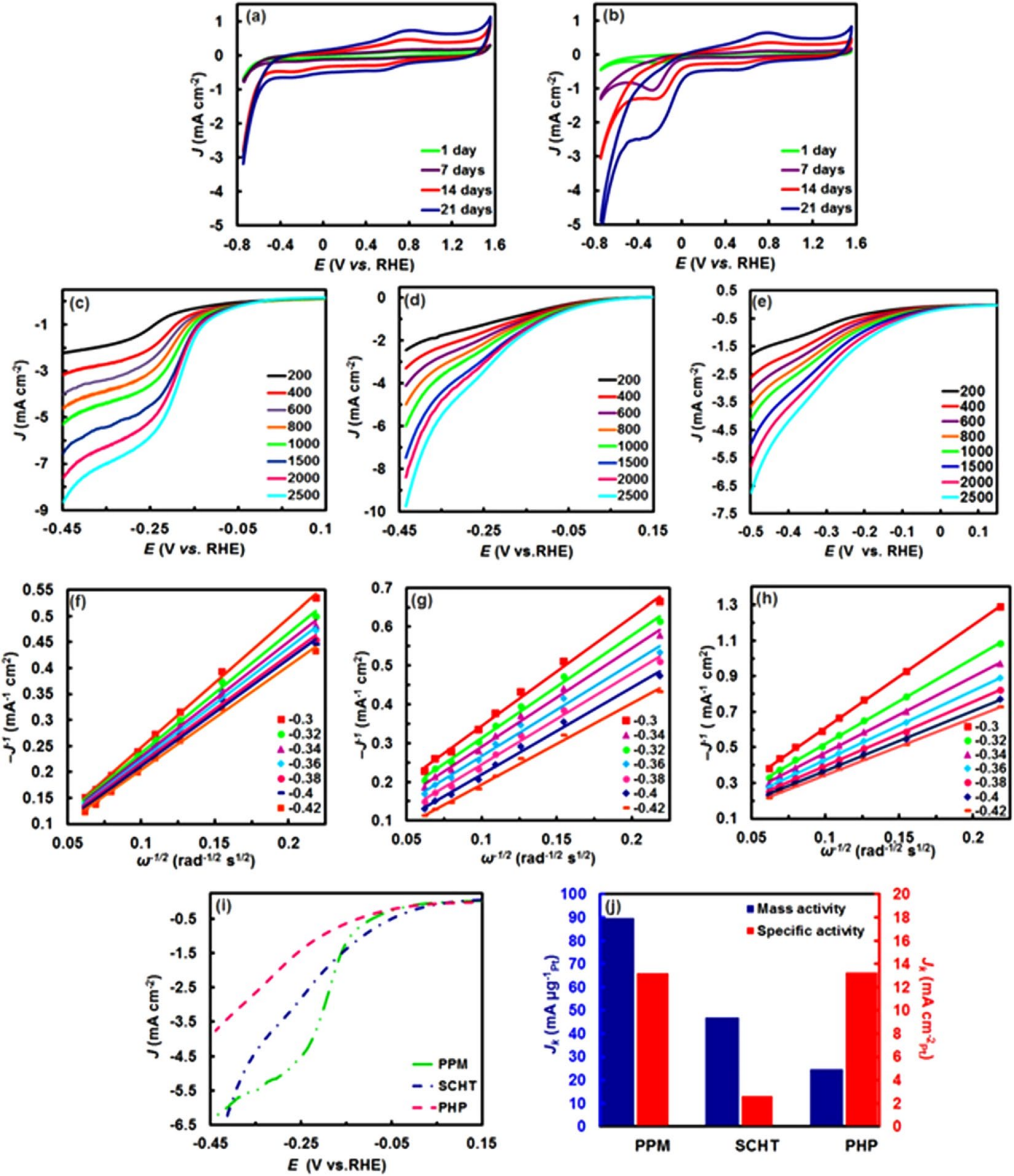
Electrochemical characterization of platinum crystals for oxygen reduction reaction. CVs of the Pt NCs obtained at various times in an (**a**) N_2_- and (**b**) O_2_-saturated 0.1 M HClO_4_ solution with the cyclic potential sweeping between −0.8 and 1.5 V versus reversible hydrogen electrode (RHE) at a scan rate of 100 mVs^−1^. ORR polarization curves of the PtNC porous microspheres (**c**), PtNC hollow tetrahedrons (**d**), and PtNC hollow polyhedra (**e**) in an O_2_-saturated 0.1 M HClO_4_ solution with a sweep rate of 10 mVs^−1^ at different rotation speeds. (**f**–**h**) Corresponding K–L plots (*J*^−1^ vs. *ω*^−1/2^) of the three types of catalysts at different potentials vs RHE. In all cases, the current densities normalized to geometric surface area of the GC rotating-disk electrode (0.0314 cm^2^). (**i**) ORR polarization curves for three types of PtNC catalysts at a rotation rate of 1500 rpm. (**j**) Mass and area-specific activities for the various PtNC catalysts measured at −0.32 V vs RHE at 295 K.

Figures 6a,b and S10 show the time evolution of CV profiles for Pt NC aggregates synthesized at 37 °C ranging from 1 day to 21 days (interval 7 days). In the O_2_- and N_2_-saturated solution, a potential region associated with the formation and reduction of Pt oxide was observed between 0.6 and 1.5 V vs. RHE, and weak featured potential region associated with hydrogen adsorption/desorption processes on the surface of crystalline Pt NC aggregates was observed between 0 and 0.37 V vs. RHE^2^ (Fig. S10). Note that the very low Pt loading on the electrode surface and the presence of protein layer on the surface of Pt NC aggregates can partly induce the sluggish proton transfer to the NC catalysts, causing less distinct features of Hb-Pt NC catalysts than that of Pt/C-based electrodes^2,57,58^.

Compared to the featureless CV profiles in a N_2_-saturated electrolyte (Fig. 6a), an increasing irreversible reduction current peak could be observed at approximately −0.2 V (vs. RHE) when the electrolyte solution was saturated with oxygen (Fig. 6b). Even though the protein layers located at the Pt NC aggregates cause a negative shift in oxygen reduction peak, compared to Pt/C, the distinctive features in the presence of O_2_ indicates high electrocatalytic activity of Pt NCs toward oxygen reduction^2,7,59,60^, which increase by increasing in the amount of Pt NC aggregates as a function of reaction time.

To further evaluate the effect of morphology on electrocatalytic performance, the reaction kinetics of oxygen reduction on the as-prepared Pt NC aggregates were studied using a glassy carbon rotating-disk electrode (RDE) in an oxygen-saturated 0.1 M HClO_4_ solution at different rotation rates (from 200 to 2500 rpm). Polarization curves for the ORR on three catalysts including prepared-Pt NCs at 67 °C known as polycrystalline porous microspheres (PPM), prepared-Pt NCs at 37 °C known as single crystalline hollow tetrahedrons (SCHT), and prepared-Pt NCs at 37 °C in the presence of NaBH_4_ known as polycrystalline hollow polyhedra (PHP) are displayed in Fig. 6c–e. As can be seen, in all voltammograms, the diffusion-limited current density increases by increasing rotation speeds; however, PPM exhibited shorter mixed kinetic-diffusion control region (between 0.01 and −0.25 V) than that of PHP (between −0.05 and −0.36 V) and SCHT (between 0.14 and −0.37 V) which confirm by a faster drop of the kinetic current with increasing potential compared to other catalysts^10^.

The number of electrons transferred (n) per O_2_ also investigated by Koutecky–Levich plots (J^−1^ vs. ω^−1/2^), as shown in Fig. 6f–h, in which for PPM, SCHT, and PHP were ~4.1, 3.7 and 2.2 in the potential range of −0.30 to −0.42 V, respectively. This means that oxygen is mainly reduced through a favored four-electron transfer pathway on platinum porous spheres and porous hollow tetrahedrons, whereas an inefficient two-electron pathway occurred on the porous hollow polyhedral aggregated Pt NCs synthesized with NaBH_4_, including various polyhedral shapes with more truncated structures, which manifest the pivotal role of shape-selective synthesis of Pt catalysts in the ORR electrocatalytic activity. This was further confirmed by comparing the rotating disk voltammograms of ORR for different catalysts at the same rotating rate of 1500 rpm (Fig. 6i). Significant morphology dependent electrocatalytic activity of NC aggregates can be clearly seen, so that overall limiting current density (e.g., at −0.32 V) increases with increasing the size- and shape-controlled nanocrystal aggregates, follow the order: PPM (−5.32 mA cm^−2^) > SCHT (−3.98 mA cm^−2^) > PHP (−2.33 mA cm^−2^), which are consistent with the results from the ORR activity observations (Fig. 6i). Furthermore, with more shape- and size-controlled synthesis, the onset potential of oxygen reduction shifts positively as SCHT (0.14 V) > PPM (0.01 V) > PHP (−0.05 V) (Fig. 6i). As seen, the onset potential of ORR on porous spheres was negatively shifted relative to hollow tetrahedrons; this may be attributed to higher surface coverage of Pt sites through a protein aggregation at high temperature, confirmed with EDX analysis (Supplementary Fig. S9), thereby increasing the barrier for proton transfers between Hb/Pt NCs and solution.

The half-wave potential, E_1/2,_ as an additional parameter permits to further assess information regarding the electroactivity during the ORR. PPM with higher Pt loading showed the most positive E_1/2_ (~−0.18), nearly 50 and 120 mV higher than SCHT (~−0.23) and PHP (~−0.3) catalysts, respectively (Fig. 6i). By comparison, although PPM exhibited a negative shift of onset potential, the current density and E_1/2_ of PMM is much better which can be due to more aggregation degree of platinum nanocluster by which the Pt reaction sites increased, improving the electrocatalytic activity of PPM as compared to PHP, thus, being the best electrocatalysts. To further kinetic study, the kinetic-limited current (J_k_), rate constant, mass activity, and specific activity were calculated and described in Table 1^7,10^. It was found that J_k_ for PPM (61.4 mA cm^−2^) is 3.1 and 6.5 times higher than that of SCHT (19.8 mAcm^−2^) and PHP (9.4 mAcm^−2^) at −0.32 V, respectively. Besides it is greater than that of previously reported Au_11_ clusters (17.9 mAcm^−2^)^59^ and Pt_n_ clusters (n = 12, 28, 60)^7,10^, confirming the strong effect of cluster aggregation. The J_K_ values for each Pt NC catalyst was almost rotation-rate independent, indicating that the expected “n” for each catalyst in oxygen reduction is valid^7^. The electrochemically active surface areas (ECSA) for SCHT, PPM, and PHP were calculated by Cu_UPD_ stripping and estimated to be 1799.4, 677.7 and 185.3 m^2^ g^−1^, respectively (Supplementary Fig. S11).

**Table 1:** Kinetic parameters for ORR using Pt NCs/GC electrodes.

Hb/Pt NCs	PPM	SCHT	PHP
number of electrons transferred	4.1	3.7	2.2
onset potential [V]	0.01	0.14	−0.05
J_k_ at −0.32 V (mA cm^−2^)	−61.38	−19.77	−9.39
rate constant (cm s^−1^)	1.29 × 10^−1^	4.62 × 10^−2^	3.68 × 10^−2^
Mass activity at −0.32 V (mA µg^−1^)	89.61	47.07	24.58
Specific activity (mA cm^−2^)_Pt_	13.22	2.62	13.26

Interestingly, we found significant enhancements in the specific ECSA for SCHT relative to PPM (~2.65 times) based on the fact that the Pt surface area is approximately inversely proportional to the particle size^61,62^. Besides, the high porosity, hollow structure, and shape selectivity endowed SCHT with outstanding higher specific ECSA compared to PHP, Pt/C (66.05 m^2^/g) and the other reported Pt-based catalytic systems^63^. Interestingly, ECSA for PPM is more than that of PHP and Pt/C thanks to a great number of electrochemically active sites in larger NC aggregates in PPM, which is outweighed the size of cluster aggregates^9,63,64^. Figure 6j shows the mass activity and the specific activity in the high overpotential region (−0.32 V versus RHE) for different Hb-stabilized PtNC aggregates. It is seen that the highest catalytic activity is achieved for the largest PtNC aggregates, so that the mass activity of PPM is 89.6 mA µg^−1^_Pt_ at −0.32 V, which is almost 1.9 times and 3.6 times higher than that of SCHT (47.1 mA µg^−1^_Pt_) and PHP (24.6 mA µg^−1^_Pt_), respectively. Moreover, the specific activity of PPM was found to be 5 times larger than that of the SCHT at −0.32 V (Table 1). Note that although increasing the size of cluster aggregates can reduce specific ECSA, the specific activity in PPM increases due to a great number of electrochemically active sites in larger NC aggregates^2,65^. This clearly indicates the significant size-dependent ORR activity upon NC aggregation by which the mass and specific activities increase by increasing the particle size, consistent with some of the previous findings for platinum nanoparticles^8,9,61,66^. This result has been described as the crystalline platinum cluster surface increased, the specific activity for ORR decreased^67^. The ratio of (100) and (111) crystal faces as electro-catalytically active sites, providing lateral adsorption and dual-site dissociation of oxygen molecules on the electrocatalyst during ORR, change as a function of the particle size, thereby resulting in a strong correlation between mass and specific activities of the ORR and the size of the Pt particle^8,68,69^.

Additionally, the type of surface facet of nanocrystals, which can be controlled by the morphology of sacrificial templates, is effective in determining the ORR activities^8,63^; so that the {111} planes are more active than {100} planes on the ORR in HClO_4_ solution^6,63^, bearing this in mind, PPM with crystalline porous spheres with broad size distribution indicates the highest ORR activity because due to having more active sites than SCHT with a narrower particle-size distribution^68^. Also, the surface oxygenated species such as hydroxyl reaction intermediates produced from water during the oxidation process^70^ decrease by increasing the coordination of surface Pt atoms when the particle size increased^9,61,66^; this may be mainly because of the changing in the potential-dependent adsorption of OH_ads_ on the crystal faces can be induced by Pt-particle size, improving the O_2_ reduction activity^61^. Moreover, decreasing fractions of H_2_O_2_ as an intermediate ORR reaction induced by complete 4-electron pathway could be an important factor in improving SA obtained for PPM^9^. On the other hand, better ORR activity of the SCHT with hollow tetrahedral structures than PHP with polyhedral shapes can be attributed to their {111} facet-dominant surfaces^8^ as well as their larger specific ECSA^63,71^. Considering lower specific activity of SCHT than that of two other catalysts (Fig. 6j), it seems that with decreasing particle size the contribution of {111} facets in the specific activity, and the concentration of aggregated clusters as active site become smaller in Pt NC aggregates. Besides, the low ORR activity of PHP can also be ascribed to the large amount of under coordinated Pt atoms on the surface of PHP due to the presence of the dendritic network morphology of PtNC aggregates having high strong oxygen binding energy^8,63^.

The metal loading on RDE was varied between 0.38 and 0.68 µg/cm^2^ for the Pt nanoclusters which are supported by inductively coupled plasma mass spectroscopic (ICP-MS) and EDS analyses^66^ (Fig. S9), whereas the Pt/C catalyst with the same loading did not show any ORR activity. These results, as well as the tremendous gains in specific and mass activities of the well-dispersed PtNC aggregates with ultra-low amount of Pt loading indicate that the ORR activity of Pt NCs was mostly due to: (i) the well-controlled size and shape of platinum nanoclusters upon aggregation-induced enhancement effect on ORR activity^66^; (ii) excellent conductivity of nanoclusters; and (iii) the use of hemoglobin as capping agent that provides shape selective NC aggregation, leading to a formation of a crystal-like cluster network with large specific surface area^63,72,73^. Note that ultra-small size of the Pt primary cluster (cluster seeds) and the high dispersion of Pt NC aggregates followed by the full utilization of the Pt crystalline surface could be paramount factors in achieving full ORR catalytic activity (preferably specific activity) on Pt^67,71^. Overall, significant voltammetric currents of well-dispersed Pt NC aggregates with ultra-low Pt loading also suggest a capability approach towards designing cheap and stable fuel cell catalysts for the ORR electrode with an excellent ECSA and mass activity, a goal of accelerating the commercialization of the fuel cell technology. It is also found that physical mixtures of Pt NCs with TiO_2_ nanoparticles results in lowering the cathode overpotential, so that the onset potential of ORR shifted positively to around 0.2 V and the peak current density increased; this indicates the promising potential for developing a better oxygen reduction reaction (ORR) catalysts using different composites of as-prepared Pt NC aggregates (Fig. S12).

In addition to reducing the Pt loading^74^, designing durable Pt-based ORR electrocatalysts is also a critical issue for the commercialization of PEM fuel cells^64^. The accelerated degradation test (ADT) to predict the durability of the prepared Pt-catalysts were conducted by comparing the ECSA before and after 1000 CV cycling in an O_2_-saturated HClO_4_ solution between 0 and 1.5 V, at 100 mV/s^6,65^ (Supplementary Fig. S13). Based on ADT for 1000 cycles, the loss of ECSA calculated from the charge associated with hydrogen adsorption indicates nearly the same results for both SCHT and PPM, i.e., about 9% loss, that is less than that of PHP (13.3%), confirming the effect of shape-controlled synthesis on improving the stability of Pt catalysts. According to the previous reports, the loss in ECSA of Pt NC aggregates is much smaller than that of commercial Pt/C catalyst^64^. Furthermore, the long-term stability of the Pt NC catalysts was performed by cyclic voltammetry in a N_2_-saturated 0.1 M HClO_4_ solution between 0 and 1.4 V (Data has not shown). Measuring H adsorption shows no change for PHP and also increased Pt surface area for PPM and SCHT, indicating no loss of Pt surface area or/and activity, while there is a large drop of 21% in ECSA after1000 cycles for Pt/C with deposited amount of 10 times more than the prepared Pt catalysts^65^.

The decrease in stability of Pt/C during the ORR is commonly related to the corrosion of carbon, which results in the detachment of Pt nanoparticles from the carbon support and Ostwald ripening/aggregation of Pt nanoparticles supported on carbon^64^, while from the results of the long-term stability and ADT tests, all of the Pt NCs aggregates showed higher stability than Pt/C. This result, which is comparable to those reported previously^64^, can be ascribed by two ways: (i), as the dominating reason, the effective coverage of the surface of Pt NC aggregates by hemoglobin with the large size (574 residues or, 64.5 kDa) as well as the special hollow porous structure of crystalline Pt NC aggregates can significantly prevents the electrooxidation and dissolution of Pt via the Pt^2+^ oxidation state; thus, the degradation of Pt catalysts is decreased, thereby maintaining crystal morphology during the stability test^6,26,65^. (ii) The strong interaction between amino groups of Hb as capping agent and COOH group-functionalized MWCNT inhibits the dissociation of tightly bound Hb/Pt NCs from electrode surface^64^. As expected, the ECSA measurements are in good agreement with the catalytic ORR activities; besides, these findings are consistent with high photostability of nanoclusters. It is required to investigate detailed mechanism of improved durability and ORR activity in future; however, the desirable obtained results indicate that the proposed novel Pt NCs platforms can act as more sustainable catalysts in fuel-cell electrochemistry and they are promising in the development enzyme-less electrodes for biofuel cell.

### Evaluation of novel fluorescent nanocrystals for cancer cell targeting imaging and cytotoxicity assay

**Figure 7:**
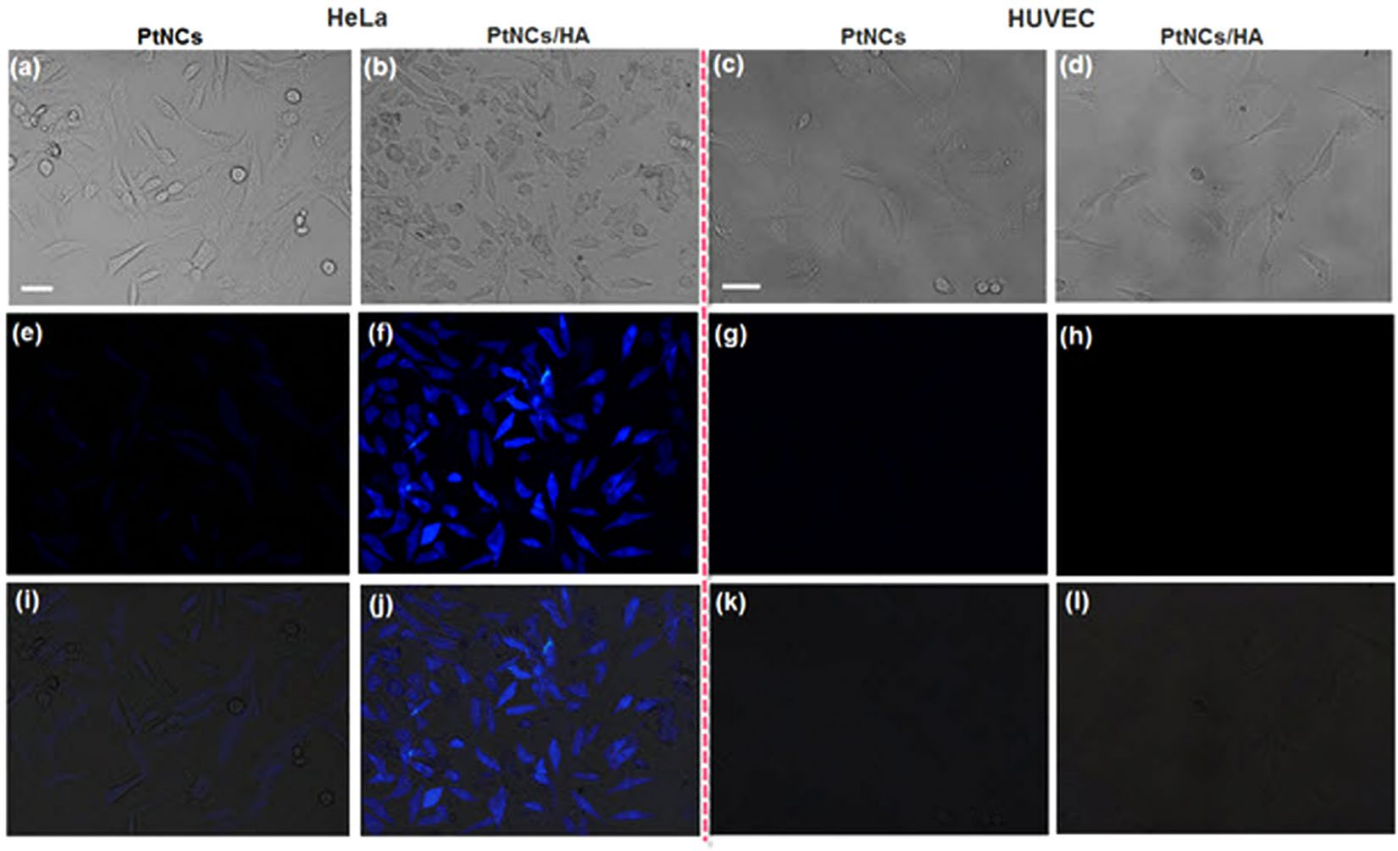
Cellular imaging. Bright-field (**a**–**d**) and the corresponding fluorescence (**e**–**h**) and merged (**i**–**l**) images of HeLa (CD44-overexpressing cell line) and HUVEC (CD44 receptor-deficient cell line) cells treated with Pt NCs and HA-conjugated Pt NCs at 6 h. Scale bar: 20 μm.

In this study, the applicability of as-prepared Pt NCs in cellular labeling and imaging, as one of the challenging areas in medical research was also investigated thanks to its excellent photostability, chemical stability, and low cytotoxicity. HeLa cells (human cervical cancer cells) as CD44-overexpressing cell line, and HUVEC cells (Human Umbilical Vein Endothelial Cells) as CD44 receptor-deficient cell line were chosen as cancer and normal cell line models, respectively. Considering overexpression of the CD44 in HeLa cells, the hyaluronic acid (HA) was used for cellular labeling which can be able to easily conjugate to the available amine group in Hb layer on the surface of Pt NCs by EDC/NHS reaction, resulting in the Hb/Pt NCs-HA nanocarrier^26,75^. FTIR analysis confirmed the successful conjugation of HA to Hb/Pt NCs using carbodiimide chemistry accompanied with the significantly reduced free carboxyl groups on the HA surface due to conjugation (Fig. S14)^26,75^. When the CD44-overexpressing HeLa cells are incubated with Pt NCs/HA, strong blue fluorescence observed inside the cells suggesting internalization of Pt NCs/HA through the HA receptor-mediated endocytosis, thereby increasing the amount of Pt NCs in HeLa cancer cells (Fig. 7f)^26,75,76^. In contrast, the fluorescence intensity from the uptake of Pt NCs without the HA was insignificant compared to Pt NCs/HA even after 6 h of incubation, probably due to non-specific interaction (Fig. 7e)^26^. Moreover, weakly blue fluorescence from the Pt NCs as well as Pt NCs/HA was observed in HUVEC cells with low expression levels of CD44 under the same conditions (Fig. 7g,h), indicating that fluorescent Hb-Pt NCs/HA nanocrystals have the ability to target specific cell types that overexpress CD44 which lead to higher endocytosis compared to the unmodified Pt NCs^26^. The above-mentioned observations are also confirmed with merged images (Fig. 7i–l).

**Figure 8:**
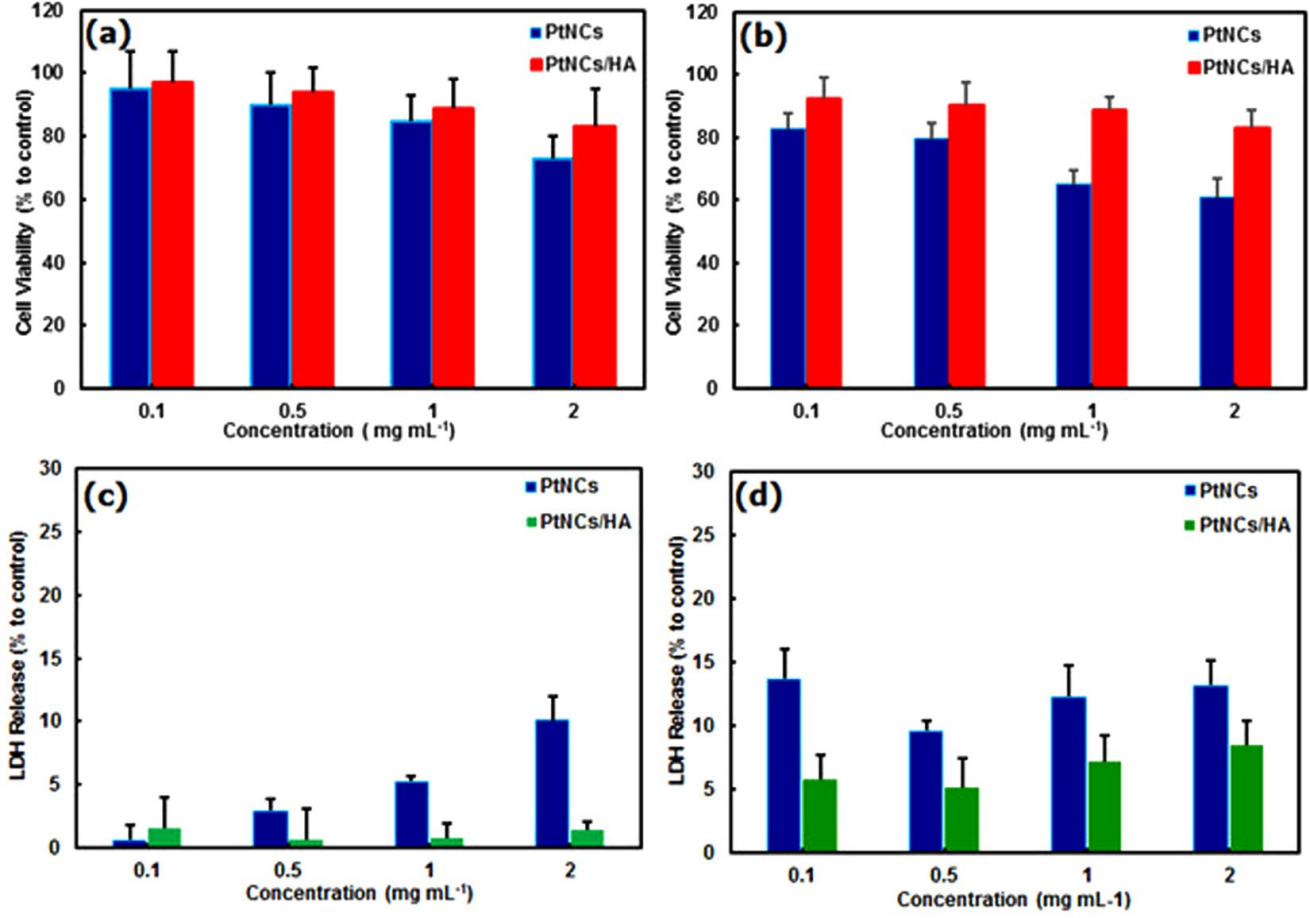
Cytotoxicity of Pt NCs and HA-conjugated Pt NCs. MTT and LDH assays (n = 3) in HeLa cells (**a**,**c**) and HUVEC cells (**b**,**d**) for 24 h.

In order to evaluate the cell viability, the MTT and LDH assays were also done for HeLa and HUVEC cells at various concentrations of the Pt NC nanoplatforms (i.e., 0.1, 0.5, 1, 2 mg mL^−1^). The results clearly demonstrated that the Pt NCs/HA are more biocompatible than Pt NCs (Fig. 8a,b)^26^. Furthermore, the LDH assay indicated that the Pt NCs/HA did not induce any membrane integrity in Hela cells compared to HUVEC cells (Fig. 8c,d). However, Pt NCs having dose-dependent LDH leakage led to good cell viability (~75% after 24 h incubation time), and low plasma membrane damages in the range of 0.1–1.0 mg mL^−1^, which can be due to the more resistant membranes of the cancer cells than the normal cells (Fig. 8c). From the above-mentioned results, it is evident that HA with easily functionalized sites, not only increases the biocompatibility of Hb/Pt NCs but also can serve as a targeting ligand to detect the CD44 receptor on the cells^26,75–77^.

**Figure 9:**
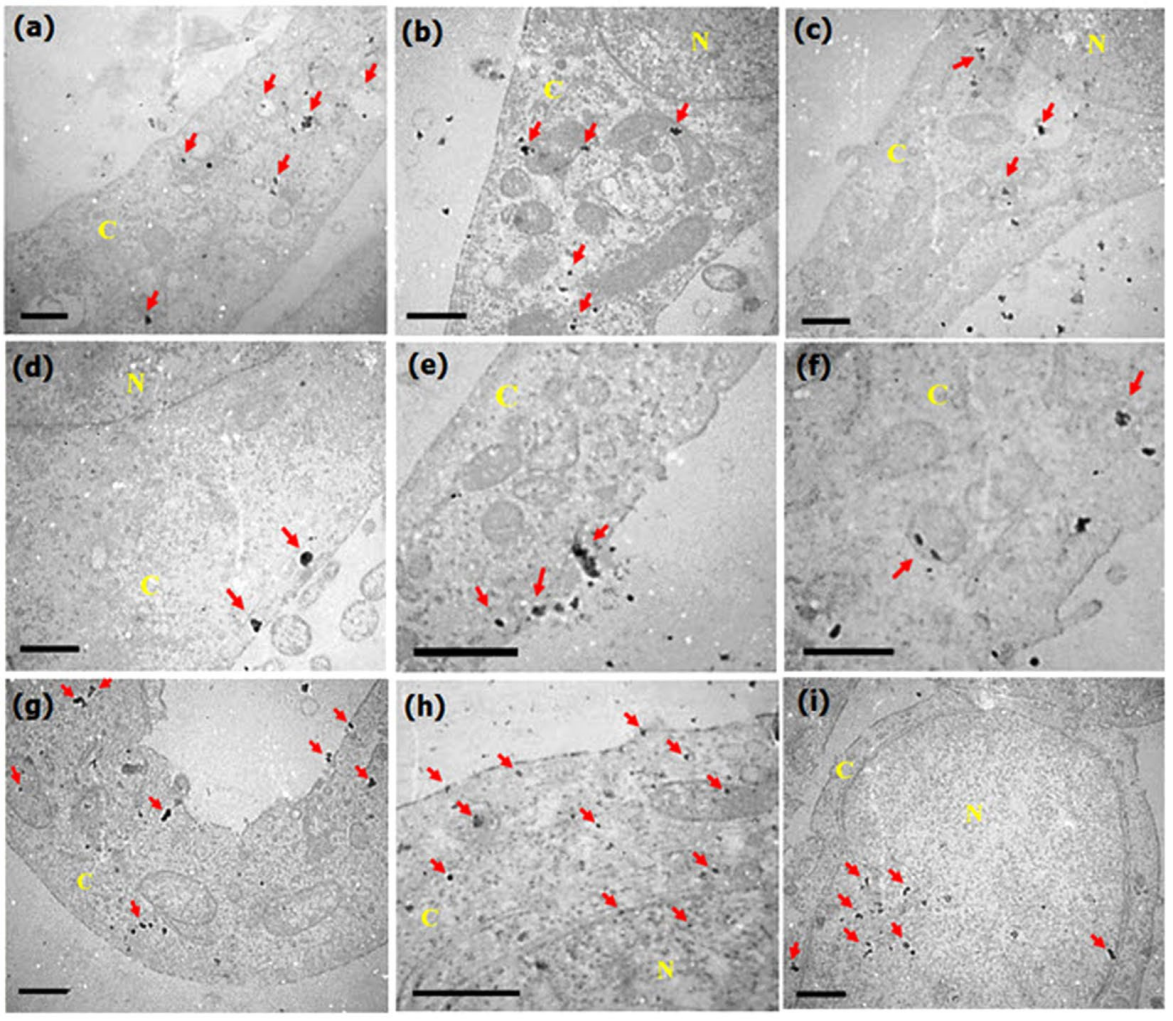
Uptake mechanism of Pt-NCs/HA. TEM images of HeLa cells treated with 1.0 mg/mL Pt NCs/HA for 1 h and 6 h. Scale bars: 500 nm for (**a**–**d**,**f**–**i**) and 1 µm for (**e**).

In order to further elucidate the uptake mechanism of Pt NCs/HA, the TEM test images of HeLa cells was taken after 1 and 6 h of incubation (Fig. 9). According to the TEM images, single or small agglomerates of Pt NCs/HA were localized in both the nucleus and the cytoplasm of cells (Fig. 9a–i); however, some of them were enclosed within endosomal vesicles (Fig. 9a–c and f–h). As expected, after 6 h of incubation, more Pt NCs were internalized compared to 1 h, at the same concentration (1.0 mg mL^−1^). During the endocytosis processes, the cells internalize extracellular molecules accompanied by temporary changes in plasma membrane integrity. This was followed by the detachment of the surface membrane invagination part and finally the formation of new intracellular vesicles. Bearing this in mind, the obtained images exhibited: (i) the presence of single tetrahedron or small agglomerates of Pt NCs in the cell membrane concomitant with changes in plasma membrane integrity (Fig. 9d,e,g,h), (ii) the endosomes enclosing the Pt NCs/HA near the plasma membrane that moves inwards of cells (Fig. 9a-c,e,f,h), and also (iii) internalized NC aggregates in HeLa cell cytoplasm which could be presumably as a result of later endocytosis (Fig. 9a,b,f,g)^78–81^. Therefore, indeed, the endocytosis is a principal uptake mechanism behind the internalization of Pt NCs/HA, which promotes the processes of receptor-mediated endocytosis due to surface attachment of HA onto the NCs. However, the diffusion process is undeniable for single particles that found freely in the cytoplasm.

In summary, the shape-controlled growth of Pt NCs using tetra-domain adult hemoglobin has been studied. Peptide chains can selectively stabilize Pt-{111} facets and hence regulate luminescent hollow nanotetrahedrons under the alkaline condition for at 37 °C. We suggest that the shape evolution of Pt cluster seeds is dictated by kinetic and thermodynamic control based on a combination of oriented attachment and Ostwald ripening processes The shape selectivity was found to decrease by increasing the reduction rate of precursor using suitable amounts of NaBH_4_ or increasing temperature by which the exclusive OA growth stage is facilitated while enhancing their luminescent intensity simultaneously; thus highly luminescent porous polycrystalline microspheres were obtained at 67 °C corresponding to the multilevel OA-based growth kinetic models. We demonstrated that the aggregation-induced emission enhancement process is one crucial point for synthesizing nanoscale Hb-directed luminescent Pt crystalline. It is believed that these luminescent crystals were produced by aggregated Pt(0)@Pt(II)– Hb core-shell structures formed by dense aggregation of oligomeric Pt(II)–X complexes which controlled by platinophilic Pt(II)···Pt(II) interactions on generated Pt(0) cores. The unique hollow porous structure of Pt crystals provides high active sites accompanied by large specific surface area; therefore, even ultra-low Pt loading (0.025 mg Pt/cm^2^) can lead to 50- to 200-fold higher mass activity and 20- to 300-fold higher specific activity compared to Pt/C and the most efficient Pt catalysts reported so far. The as-prepared Hb-Pt NCs also exhibited high potential to use in cellular labeling and imaging thanks to the excellent photostability, chemical stability, and low cytotoxicity.

## Experimental Section

### Synthesis of hemoglobin-capped Pt NCs

Platinum NCs were synthesized by mixing 2.5 mL of Hb (0.11 mM) and 2.5 mL of H_2_PtCl_6_ at different molar ratios of Pt: Hb from 0.56:1 to 18.2:1 under vigorous stirring at 37 °C. Sampling of the reaction solution was done at various stirring times.

### Structural characterization

Fluorescence measurements and UV/Vis absorption were performed using a Perkin-Elmer LS-50B fluorescence spectrometer (Perkin-Elmer, UK), and Model Scinco UV S-2100 (Cinco, Korea), respectively. The TEM and HRTEM images were recorded using an EM10C Zeiss transmission electron microscope (Zeiss, Germany) and a Philips CM30 transmission electron microscope (Philips, Netherlands) with accelerating voltages of 80 and 300 kV, respectively. The XPS measurements were carried out on an ESCALab220I-XL spectrometer (VG, U.K.) with monochromatized Al Kα radiation at 1486.6 eV, operating at a vacuum <10^−7^ Pa. MALDI MS analysis of Hb and Hb/Pt NCs were conducted using a Kratos Axima CFRplus (Shimadzu Biotech, Manchester, U.K.). Dynamic light scattering (DLS) and ζ-potential were determined using a Zetasizer Nano ZS (Malvern Instruments Ltd., U.K.) equipped with a 633 nm (He–Ne) laser.

### Electrochemical characterization

Electrochemical measurements were carried out with the PalmSens Potentiostat/Galvanostat/EIS (Netherlands). A typical three-electrode system was employed, consisting of a MWCNT modified glassy carbon electrode as the working electrode, Ag/AgCl (3 M KCl) reference electrode and plain Pt sheet (1 cm^2^) as a counter electrode in 0.1 M HClO_4_. Rotating disc electrode measurement (RDEs) was carried out in an O_2_-saturated 0.1 M HClO_4_ solution at various rotation rates. Pt loading was calculated based on ICP-AES.

### *In vitro* cellular imaging and cytotoxicity

Hela human breast cancer cell line as HA receptor-positive and Human Umbilical Vein Endothelial cell line as HA receptor-negative were used to investigate the uptake of as-obtained Pt NCs and Hb-Pt NCs/HA. Cells were seeded on a six-chamber glass slide at 1 × 10^5^ cells/well with 2 ml culture medium (PRMI medium with 10% fetal bovine serum (FBS) and 1 wt. % of penciling-streptomycin) in a humidified 5% CO_2_ incubator atmosphere at 37 0 C incubator, after 24 h, the culture medium was abandoned and cells were treated with free Pt NCs, and Hb-Pt NCs/HA (with an equivalent concentration of Pt NCs), followed by incubation at 37 °C in a humidified 5% CO_2_ atmosphere for 6 h. After that, culture media were discarded, and the cells were washed with buffer (PBS 0.01 M, pH 7.4) three times to remove any unbound Hb-Pt NCs or Hb-Pt NCs/HA before fluorescence imaging. The fluorescence images were captured by an Olympus IX-81 fluorescent microscope (Olympus Imaging System, Japan). *In vitro* cytotoxicity investigation of Hb-Pt NCs and Hb-Pt NCs/HA was done for both HEK239 and Hela cell lines using the MTT method.

### LDH assay

Lactate dehydrogenase (LDH) is an enzyme widely present in cells that converts lactate into pyruvate. We used LDH assay kit (*Pars Azmun*, Iran) to evaluate plasma membrane integrity of HeLa and HUVEC cells. Briefly, 10^4^ cells/well were seeded in 96-well plate and allowed to attach overnight. Then, the cells were treated in a wide variety of concentrations (i.e., 0.1, 0.5, 1, 2 mg mL^−1^) of Pt NC nanoplatforms for 24 h. After that, the cells were centrifuged at 4500 rpm for 10 min to get the cell culture media. The cells cultured in DMEM medium without Pt NCs were used as a negative control. The LDH maximum leakage control (positive control) was prepared by adding 10 μL of lysis solution to the HeLa and HUVEC cells, 60 min prior to centrifugation. Then 10 μL of supernatant from each well was transferred to a new 96-well plate. After that 4-volume unit R_1_ buffer relative to 1 volume unit R_2_ buffer were mixed to all wells and incubated less than 5 min at 37 °C. Absorbance was measured by a microplate reader (MQX200 uQuant, Biotek, USA) at 340 nm for 3 min at 1 min intervals. LDH leakage was expressed based on the following percentage of Equation (1) ^80^. OD blank was the OD of the culture medium without HeLa and HUVEC cells.

1 $${\rm{LDH}}\,{\rm{release}}( \% )=\frac{({\rm{OD}}\,{\rm{test}}+{\rm{OD}}\,{\rm{blank}})}{({\rm{OD}}\,{\rm{positive}}-{\rm{OD}}\,{\rm{blank}})}\times 100 \% $$

## Electronic supplementary material

Supplementary Information
