# Supplementary material for: Shape-Controlled Synthesis of Luminescent Hemoglobin Capped Hollow Porous Platinum Nanoclusters and their Application to Catalytic Oxygen Reduction and Cancer Imaging
Source: Sci Rep. 2018 Sep 28;8:14507. doi: 10.1038/s41598-018-32918-w (PMC6162304; doi:10.1038/s41598-018-32918-w)
Supplement: Supplementary file 1 — Supplementary Information [file 41598_2018_32918_MOESM1_ESM.docx]

Supplementary Information for;

**Shape-Controlled Synthesis of Luminescent Hemoglobin Capped Hollow Porous Platinum Nanoclusters and their Application to Catalytic Oxygen Reduction and Cancer Imaging**

Fatemeh Molaabasi^1,2,*,¥^, Morteza Sarparast^3,¥^, Mojtaba Shamsipur^4,*^, Leila Irannejad^2^, Ali Akbar Moosavi-Movahedi^5^, Abouzar Ravandi^6^, Behnam Hajipour Verdom^7^, Reza Ghazfar^3^

^1^ Department of Biomaterials and Tissue Engineering, Breast Cancer Research Center, Motamed Cancer Institute, ACECR, Tehran, Iran.

^2^ Department of Chemistry, Faculty of Basic Sciences, Tarbiat Modares University, Tehran, 14115-175, Iran.

^3^ Department of Chemistry, Michigan State University, East Lansing, Michigan, 48824-1322, United States.

^4^ Department of Chemistry, Faculty of Basic Sciences, Razi University, Kermanshah, Iran.

^5^ Institute of Biochemistry and Biophysical Chemistry University, Tehran University, Tehran, Iran.

^6^ Department of Chemistry, Faculty of Basic Sciences, Sharif University of Technology, Tehran, Iran.

^7^ Department of Biophysics, Faculty of Biological Sciences, Tarbiat Modares University, Tehran, 14115-154, Iran.

* Fatemeh Molaabasi, E-mail: [molaabasi.fatemeh@yahoo.com](mailto:molaabasi.fatemeh@yahoo.com)

* Mojtaba Shamsipur, E-mail: [mshamsipur@yahoo.com](mailto:mshamsipur@yahoo.com)

^¥^ Fatemeh Molaabasi and Morteza Sarparast contributed equally to this work.

**Supplementary Information Captions**

***Contents***

1. Formation of hemoglobin protected Pt clusters. S5

1.1. Optimization and investigation the effective kinetic parameters S5

1.2. MALDI-TOF S10

1.3. Hg^+2^ and Cysteine tests to prove core-shell structure of Pt NCs S11

2. Shape controlled synthesis of Hb-Pt NCs upon aggregation-induced emission. S11

3. Formation Mechanism of the Pt NCs Aggregates. S13

More information regarding formation mechanism of Pt NCs S15

4. Electrocatalytic performance of Pt NC aggregates. S18

5. Cancer cell targeting imaging and cytotoxicity assay S22

6. Experimental section S22

6.1. Materials. S22

6.2. Synthesis of hemoglobin-capped Pt NCs. S24

6.3. Synthesis of HA-Conjugated Pt NCs. S24

6.4. Structural Characterization. S24

6.5. Electrochemical Characterization. S26

6.6. In vitro cellular imaging and cytotoxicity. S29

7. References: S30

***Figures***

Figure S1. UV−vis and emission spectra of as-prepared Pt nanoclusters with different molar ratios of Pt/Hb: (a, a׳) 0.56 with λ_ex_/λ_em_= (360 nm)/(455 nm); (b, b׳) 1.13 with λ_ex_/λ_em_= (360 nm)/(450 nm); (c, c׳) 2.27 with λ_ex_/λ_em_= (360 nm)/(455 nm); (d, d׳) 4.54 with λ_ex_/λ_em_= (360 nm)/(420 nm); (e, e׳) 6.82 with λ_ex_/λ_em_= (360 nm)/(420 nm); (f, f׳) 9.10 with λ_ex_/λ_em_= (360 nm)/(415 nm); (g, g׳) 13.6 with λ_ex_/λ_em_= (360 nm)/(415 nm); (h, h׳) 18.2 with λ_ex_/λ_em_= (360 nm)/(415 nm). (i) UV−vis spectra of as-prepared Pt nanoclusters with different molar ratios of Pt/Hb at the same Hb concentration (0.11 mM)…………………………………………………………………………S6

Figure S2. (a) Emission and (b) UV−vis spectra of Hb/Pt NCs collected at different temperatures (λ_ex_= 320 nm) with 0.11 mM Hb and 0.125 mM H_2_PtCl_6_ at pH∼12.4…………………………….S7

Figure S3. (a) Emission and (b) UV−vis spectra of Hb/Pt NCs obtained from monitoring reaction times with 0.11 mM Hb and 0.125 mM H_2_PtCl_6_ at 67ᵒC (λ_ex_= 320 nm)………………………….S8

Figure S4. (a) Emission and (b) UV−vis spectra of Pt NCs obtained from monitoring reaction times with 0.11 mM Hb and 0.125 mM H_2_PtCl_6_ at 37ᵒC (λ_ex_= 320 nm) upon addition of NaBH_4_ (1mL of 40 mM)…………………………………………………………………………………S9

Figure S5. Emission spectra of the Pt NCs upon addition of 100 µM (a) Hg and (b) Cys (λ_ex_/λ_em_= (320 nm)/(450 nm)……………………………………………………………………………..S11

Figure S6. Uv-vis spectra of Hb (red) and as-prepared Hb/Pt NCs (blue) after 22 days of reaction with 0.11 mM Hb and 0.125 mM H_2_PtCl_6_ at 37ᵒC……………………………………………..S11

Figure S7. TEM images of the Pt NCs obtained from mixtures of 0.11 mM BSA and 0.125 mM H_2_PtCl_6_ at 37 ºC…………………………………………………………………………………S12

Figure S8. (a) FTIR spectra of Hb (black) and as-prepared Hb/Pt NCs (red) after 22 days of reaction. (b) Far-UV circular dichroism (CD) spectra of Hb (blue) and Hb/PtNCs (red)……….S14

Figure S9. EDS spectra of Hb/PtNCs prepared at (a) 67ᵒC (b) 37ᵒC and (c) 37 °C by using NaBH_4_ (1cc of 40 mM). Insets show EDS and ICP analyses of as-prepared Hb/PtNCs………………..S19

Figure S10. CVs of the Pt NCs obtained at various times in an (a) N_2_- and (b) O_2_-saturated 0.1M HClO_4_ solution with the cyclic potential sweeping between 0 and 1.5V versus reversible hydrogen electrode (RHE) at a scan rate of 100 mVs^-1^……………………………………………………S20

Figure S11. Cu_UPD_ stripping measurements of the PtNC hollow tetrahedra (a), PtNC porous microspheres (b), and PtNC hollow polyhedra (c) in solution of 0.5 M H_2_SO_4_ + 1 mM CuSO_4_ at 20 mV/s…………………………………………………………………………………………S20

Figure S12. CVs of TiO_2_ and PtNCs + TiO_2_ in an O_2_-saturated 0.1M HClO_4_ solution with the cyclic potential sweeping between -0.2 and 1.0 V versus reversible hydrogen electrode (RHE) at a scan rate of 100 mVs^-1^………………………………………………………………………..S21

Figure S13. CVs of the PtNC porous microspheres (a), PtNC hollow tetrahedra (b), and PtNC hollow polyhedral (c) obtained before (black) and after (red) 1000 potential sweeps vs. RHE in an O_2_-saturated 0.1M HClO_4_ solution with a sweep rate of 100 mVs^-1^……………………………S21

**Figure S14.** FTIR spectra of HA (blue) and Pt NCs/HA (red)………………………………S22

# Formation of hemoglobin protected Pt clusters.

## Optimization and investigation the effective kinetic parameters

In this study, hemoglobin (Hb) was used as both reducing and capping agent for direct reduction of Pt^4+^ ions that results in synthesis of Pt NCs under one-step “green” process; typically, Pt NCs are prepared by mixing H_2_PtCl_6_ and Hb solution at 37 ºC for 10 min, followed by NaOH addition (pH ~ 12). As shown in Figure. 1a, under room light, in the absence of Pt, the Hb solution is red color and after addition of Pt immediately turns brownish color and then with the addition of NaOH, the blackish green color was obtained for molar ratios Pt: Hb from 0.5:1 to 2.5:1 and the mixed solution give brownish color for molar ratios Pt: Hb from 4.5:1 to 18.2:1, indicating the formation of platinum nanoparticles with increasing molar ratios Pt:Hb in accordance with previously reported results^1^. The rapid color change observed in the presence of Pt indicates the reaction evolved very fast (Figure. 1a). The time dependent absorption and photoluminescence emission spectra of Hb/Pt NCs were investigated for 5 days to explore the optimal Pt/Hb ratio and consequently, to control the size for preparing the highly fluorescent Pt nanoclusters (Figure. S1a-h). When solutions with different molar ratios of Pt/Hb are excited with the variation of the excitation wavelength from 350 to 415 nm, the maximal emission was observed with an excitation at 360 nm (data not shown). These different color emissions resulted from the different excitation were consistent with the formation of a mixture of Pt NCs varying in size^2^. Also, the fluorescence (λ_ex_ = 360 nm) and absorption peaks gradually increase with a stirring time up to 5 days (Figure. S1a-h, a′-h′). As seen, compared to the emission peak (λ_em_,_max =_ 450 nm) of ratios Pt:Hb from 0.6:1 to 2.5:1, the emission maxima of the Pt NCs produced from 4.5:1 to 18.2:1 showed more than 30 nm blue shift indicating the presence of smaller Pt NCs ( Figure. S1a´-h´). The low fluorescent intensities with increasing molar ratio Pt: Hb from 2.5:1 to 18.2:1 were mainly due to the ineffective protection of template protein, allowing the formation of large


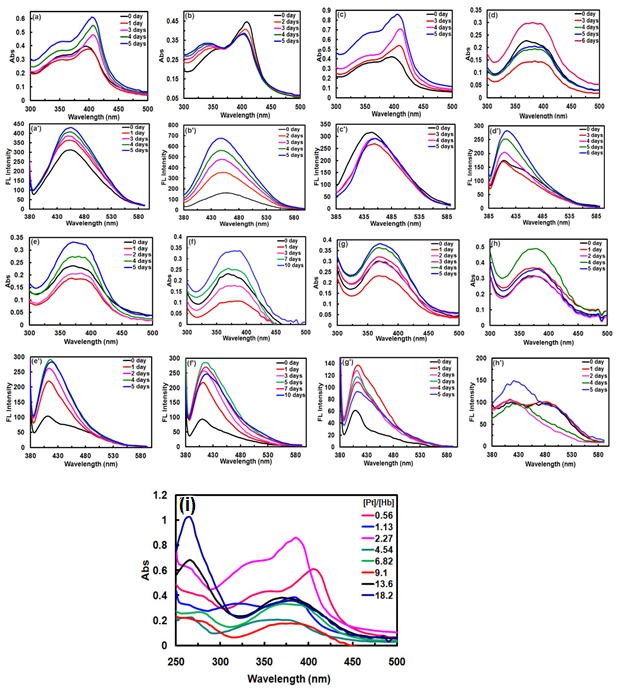


Figure S1. UV−vis and emission spectra of as-prepared Pt nanoclusters with different molar ratios of Pt/Hb: (a, a׳) 0.56 with λ_ex_/λ_em_= (360 nm)/(455 nm); (b, b׳) 1.13 with λ_ex_/λ_em_= (360 nm)/(450 nm); (c, c׳) 2.27 with λ_ex_/λ_em_= (360 nm)/(455 nm); (d, d׳) 4.54 with λ_ex_/λ_em_= (360 nm)/(420 nm); (e, e׳) 6.82 with λ_ex_/λ_em_= (360 nm)/(420 nm); (f, f׳) 9.10 with λ_ex_/λ_em_= (360 nm)/(415 nm); (g, g׳) 13.6 with λ_ex_/λ_em_= (360 nm)/(415 nm); (h, h׳) 18.2 with λ_ex_/λ_em_= (360 nm)/(415 nm). (i) UV−vis spectra of as-prepared Pt nanoclusters with different molar ratios of Pt/Hb at the same Hb concentration (0.11 mM).

sized platinum nanoparticles^3^ (Figure S1c**׳**-h**׳**). Meanwhile, from the UV-Vis spectra for 5 days shown in Figure S1i, the absorbance of PtCl_6_^2-^ at 260 nm was decreased with lowering molar ratio of Pt/Hb (18.2 to 13.6) and finally disappears for molar ratios from 9.1 to 0.6, thereby resulting in the red shift and increase of the fluorescence peak of Pt clusters^2, 4^. Also, for molar ratios from 18.2 to 4.5, one broad absorption peak was seen at ~360 nm, however, two absorption peaks centered at ~ 330 and 406 nm have been observed when the molar ratios are from 0.6 to 2.2 (Figure S1i). Thus, the Pt-to-Hb in luminescent Pt NCs is critical synthesis parameters.


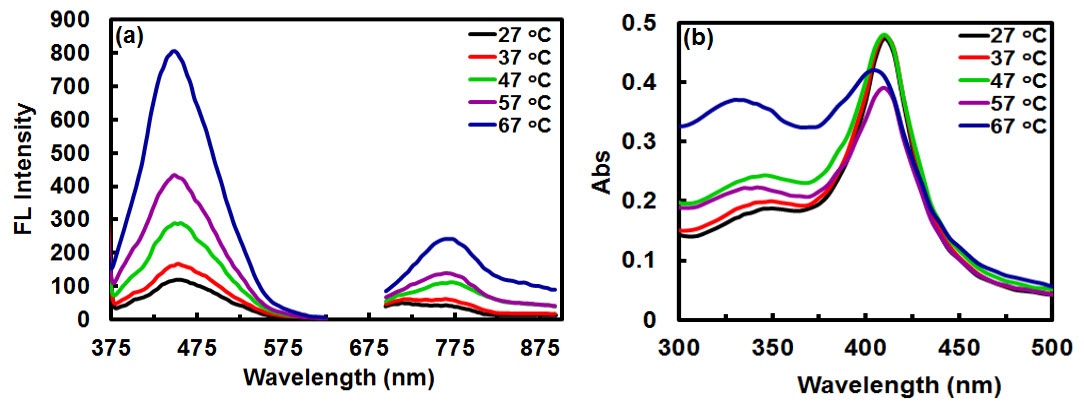


Figure S2. (a) Emission and (b) UV−vis spectra of Hb/Pt NCs collected at different temperatures (λ_ex_= 320 nm) with 0.11 mM Hb and 0.125 mM H_2_PtCl_6_ at pH∼12.4.

For the Pt/Hb ratio of 1.14, Hb/ Pt NCs showed the most intense fluorescence at λ_em_ = 450 nm in alkaline reaction media (pH~ 12.4), but not reached the equilibration after 5 days, thus the time evolution of fluorescence and Uv-vis spectra for clusters was followed from 1 h to 25 days and it appears that the reaction was nearly completed in about 22 days (Figure 1c, d). Moreover, compared to gold NCs prepared under the same conditions^5^ Hb/Pt NCs exhibit near-infrared (NIR) fluorescence emission at λ_em_ = 760 nm (λ_ex_ = 320 nm) attributed to a larger cluster size^1^. The emission intensity at 760 nm as well as 450 nm reaches the maximum until about 22 days (Figure 1d). Thus, the solution has two species, a red emitting cluster and the blue emitting Hb/Pt NCs system, when Pt NCs were synthesized with low concentration of Pt (0.125 mM). The results are shown in Figure 1c, d. Since PL band at 760 nm probably arises from the surface state^6-8^, the emission at 450 nm is much higher than that of 760 nm. This finding could be due to a low fraction of Pt (II) species on the cluster surface as discussed in XPS spectra, which is in contrast to the emission spectra of Cu NCs in the previous reports^8-9^. The large Stoke’s shift (440 nm) suggests that the NIR (760 nm) emission from aggregated Pt(II)−X complexes was mainly phosphorescence and could be attributed to metal-centered triplet excited state considering the ligand-to-metal charge transfer (LMCT) or LMMCT behavior from Pt(II)-Hb complexes (the oxygen and nitrogen atom in the amino acids to the Pt(II) ions) to the Pt atoms and subsequent radiative relaxation which can be enhanced by Pt–Pt interactions^7-14^.


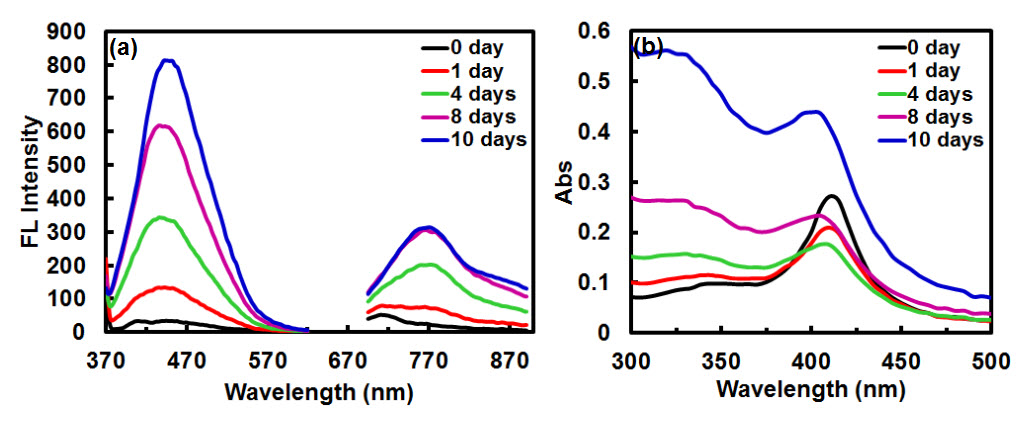


Figure S3. (a) Emission and (b) UV−vis spectra of Hb/Pt NCs obtained from monitoring reaction times with 0.11 mM Hb and 0.125 mM H_2_PtCl_6_ at 67ᵒC (λ_ex_= 320 nm).

Owing to the need for long time synthesis of highly luminescent Hb-Pt NCs, two methods were employed: (i) formation in different temperature, and (ii) using NaBH_4_ as a strong reducing agent^15-16^. The effect of temperature on the reaction rate was investigated over the range from 27 to 67 ºC for 24 h, and fluorescence and Uv-vis spectra were recorded (Figure S2). As can be seen in Figure S2a, fluorescence intensity is increased with temperature indicating the higher temperature is, the more amounts of nanoclusters will be formed, whereas maximum PL intensity achieved at elevated temperature of 67 ºC. Investigation of time evolution of PL intensity during 10 days also shows gradually fluorescence enhancement (Figure S3a).

Furthermore, fluorescent enhancement was achieved in the presence of NaBH_4_ for 10 days (Figure S4a). For this reason, Pt clusters were synthesized by reducing H_2_PtCl_6_ (2.5 mL, 0.125 mM) with NaBH_4_ (1 mL, 40 mM) in the presence of Hb (2.5 mL, 0.125 mM) and NaOH (0.5 mL, 1.0 M; Figure S4). Under vigorous stirring, NaBH_4_ was added slowly to the mixture within 10 min and allowed it to stir continuously at 37 ºC for 10 days. The color of the solution evolved from red to light brown and finally to clear yellow. The heme degradation under formation of small platinum nanoparticles (Pt NPs) was indicated by the change of solution color from red to a pale yellow, and the absence of Soret band in near-UV (400-436 nm) (Figure S4b).


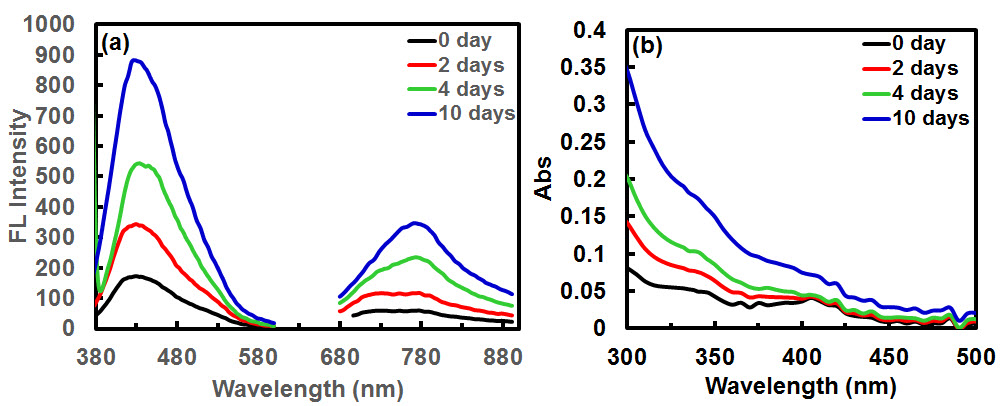


Figure S4. (a) Emission and (b) UV−vis spectra of Pt NCs obtained from monitoring reaction times with 0.11 mM Hb and 0.125 mM H_2_PtCl_6_ at 37ᵒC (λ_ex_= 320 nm) upon addition of NaBH_4_ (1mL of 40 mM).

The mixture showed a high intensity of fluorescence emission maxima at 420 nm, with a ~35-40 nm blue-shift in comparison with that synthesized in the absence NaBH_4_, illustrating of smaller core sizes^17-18^ (Figure S4a). Quantum yield was calculated relative to quinine sulfate as the standard at optimized condition for Pt NCs synthesized at 37 ºC, 67 ºC and in the presence of NaBH_4_ at 37 ºC and 0.7 (21 days), 2.8 (10 days), and 3.4 % (10 days) was obtained, respectively^5, 19^. This indicates that temperature and NaBH_4_ as strong reducing agent affect reaction kinetics resulted in higher cluster formation and consequently higher quantum yield.

## MALDI-TOF

The clusters composition as a significant factor in determining the chemical and physical properties of these nanoplatforms, was elucidated by matrix assisted laser desorption ionization-time of flight (MALDI-TOF) experiments. As shown in the Figure 1e, free Hb sample at alkaline pH was composed of two distinct peaks at m/z 15 296 and 16 003 Da corresponding to α-chain (MW: 15 125 Da) and β-chain (MW: 15 866 Da) of hemoglobin, respectively^17, 20^. While the peak intensities were decreased upon cluster formation, the α-chain peak was shifted to 16 466.5 and 18 416.5 Da, and the β-chain peak to 17 170.5 and 19 123.5, respectively. Mass difference from the lower mass peaks (~ 1170.5 Da) are assigned to Hb/Pt_6_ NCs and from the higher mass peaks (~ 3120.5 Da) are ascribed to Hb/Pt_16_ NCs, however in both cases the quantity of the former was higher than the latter. This implies that two clusters are produced using Hb as template, which is in perfect agreement with the PL measurements consist of the strong blue emission related with Pt_6_ NCs and the weaker red emission related with Pt_16_ NCs. Additionally, the reduced mass spectral intensity with a substantial broadening of the features in Hb-Pt NCs than free Hb, the fact that Pt NCs may be able to alter protein structure leading to better cluster formation (Figure 1e)^17, 21^.

## Hg^+2^ and Cysteine tests to prove core-shell structure of Pt NCs


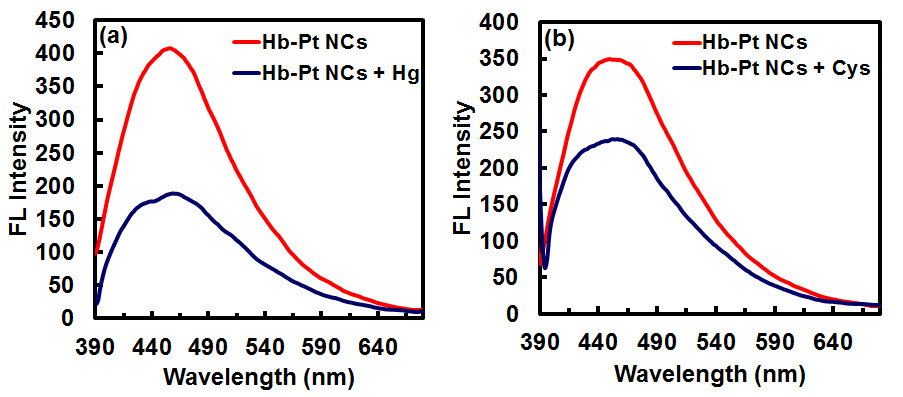


Figure S5. Emission spectra of the Pt NCs upon addition of 100 µM (a) Hg and (b) Cys (λ_ex_/λ_em_= (320 nm)/(450 nm).

# Shape controlled synthesis of Hb-Pt NCs upon aggregation-induced emission.


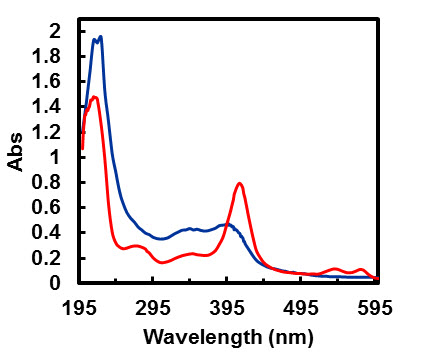


Figure S6. Uv-vis spectra of Hb (red) and as-prepared Hb/Pt NCs (blue) after 22 days of reaction with 0.11 mM Hb and 0.125 mM H_2_PtCl_6_ at 37ᵒC.

**Note:** The absorption spectrum of the native-state structure of the Hb shows a number of distinct bands centered at 349 (ε band), 540, and 575 nm (oxy-band or Q-band)^22^ (Figure. S7). The Q-band position reflects microenvironment changes of heme in Hb^23^. The intensity changes of the tyrosine/Trp band (285nm), ε band (349nm), Soret band, and also the Q-band (575 nm) indicates that nanocluster aggregates induced by the Pt NCs formation, can access both the heme and the Trp residues^24-25^, and thus this may affect the conformation of hemoglobin as capping agent in the Hb/Pt NCs system.


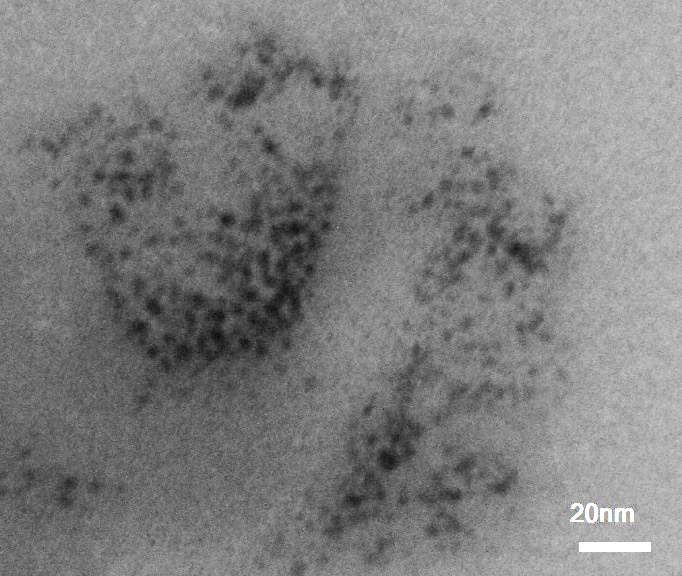


Figure S7. TEM images of the Pt NCs obtained from mixtures of 0.11 mM BSA and 0.125 mM H_2_PtCl_6_ at 37 ºC.

# Formation Mechanism of the Pt NCs Aggregates.

To study the formation mechanism of the Pt NCs in the Hb aqueous solution, the FT-IR spectra of pure Hb and Hb/Pt NCs solutions were determined (Figure S8a). The FTIR spectra provide information on the secondary structural change before and after metal nanocluster encapsulation, because the amide bands are highly sensitive to environmental change, especially, amide I mainly C=O stretching (1600 –1680 cm^−1^), and amide II band arising from N–H bending (60%) and C–N stretching (40%) (1500 –1620 cm^−1^). The band appearing at ~2960 cm^−1^ is due to saturated C–H stretching vibrations. Other bands are those at ~ 3421 cm^−1^, which can be assigned to the stretching vibration of N–H of amide group (ca. amide A′) and that at ~1395 cm^−1^ is due to C–N stretching vibration of aromatic amine. Compared to the IR spectra of native protein, the IR spectra of the Hb/PtNCs showed several distinct changes. After the formation of Pt NCs, the shape and peak position of the amide I band (1658 cm^−1^) of Hb is nearly the same and decreased, but amide II (1533 cm^−1^) and aromatic amine band (1395 cm^−1^) present in free Hb are disappeared in Hb/PtNCs with appearance of a new band located at 1384 cm^−1^. Tyrosine is a very strong IR absorber, which over dominated the amide II bands. The obvious changes for amid II band and the aromatic amine band responsible for the binding of platinum ions with protein via free amine groups and the decreasing in intensity of amide I and amide A′ bands suggest that there is a substantial change in the conformation of the Hb from the free state, i.e. fewer helical structures are present as a result of interaction with the Pt NCs^26^. In addition, the preparation of Pt NCs at high pH leads to the enhancement of the peak intensity centered at 1384 cm^−1^ which could correspond to the vibration of tryptophan (Trp). XPS results in the literature also indicate that the thiol-containing amino acids were effective for the synthesis PtNCs resulting in controlled AIEE phenomenon confirmed by photoluminescence spectra, UV-Vis absorbance and TEM pictures. Therefore, it can be concluded that the binding interactions due to hydroxyl (i.e., tryptophan, threonine, tyrosine, and serine), thiol (i.e., cysteine) and amine (i.e., histidine, lysine, and arginine) groups as excellent nucleating agents are probably responsible for the resulting Hb/Pt NCs nanocrystals formation^27^.


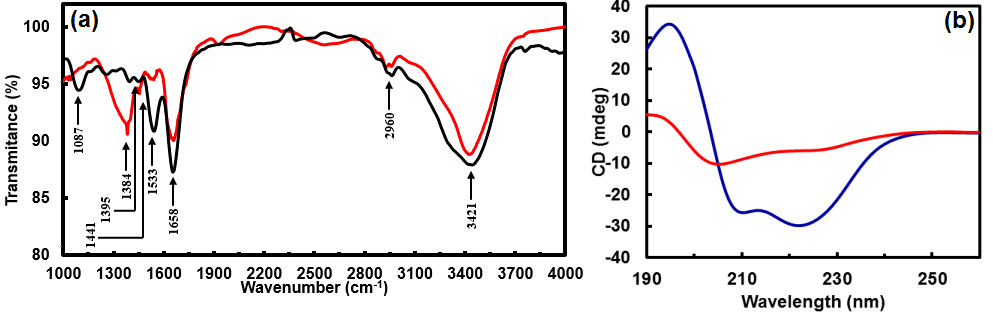


Figure S8. (a) FTIR spectra of Hb (black) and as-prepared Hb/Pt NCs (red) after 22 days of reaction. (b) Far-UV circular dichroism (CD) spectra of Hb (blue) and Hb/PtNCs (red).

For a more thorough elucidation of the formation mechanism of 3D Pt nanotetrahedra, the conformational behavior of Hb in Hb/PtNCs was investigated by circular dichroism (CD) measurements. Figure S8b shows a comparison of the Far-UV CD spectra of free Hb and Hb/PtNCs. The free Hb shows a positive CD at 195 nm and two negative CD bands at 208 nm and 222 nm, which are characteristics of a high α-helical content. We calculated the secondary structural elements using the J-700-Standard Analysis program and found that the incorporation of Pt NCs resulted in a 47% decrease in α- helical (i.e. mean residual ellipticity at 222 nm), and 42% and 18% increase in β-sheet and random coil (i.e. mean residual ellipticity at 195 and 200 nm) structures, respectively. Meanwhile, after the formation of Pt NCs, the negative band at 208 nm present in CD spectrum of the free Hb is shifted to 205 nm. As it is known, the conformation of protein must undergo a certain degree of changes to facilitate the effective formation of clusters. α-Helix has 3.6 amino acids per turn of the helix and β-sheet structure can be seen as a kind of special α-helix only with two amino acid residues through stretching. α-Helix are formed when the carbonyl O of the i ^th^ amino acid bonds to the amide H of the i ^th^ + 4 aa (4 amino acids away). Therefore, H bonding is the main factor to maintain the α-helix structure of the protein.

### More information regarding formation mechanism of Pt NCs

Particles generated as a function of time during the four-stage growth process with uniform, narrow size/shape distribution confirmed this. Moreover, an increase in the zeta potential with time due to electrostatic repulsion increasing between the particles synthesized under high pH condition, can allow controlling aggregation kinetics and thus the formation of hollow clusters as shown in Figure 2d^28^. Since, PL peak of nanoclusters is not shifted during aggregation, therefore, it can be hypothesized that Pt nanoparticles grow mainly via Pt_6_ and Pt_16_ clusters rather than clusters of other sizes. Thus, Pt_6_ clusters play an important role as an elementary cluster in the formation of the Pt nanoparticles. As a result, the as-prepared porous hollow nanotetrahedrons of Pt NCs were obtained using the combined process of both oriented attachment and Ostwald ripening including the following four consecutive steps: (i) production of Pt nanoclusters (ii) random aggregation of Pt NCs to produce primary irregular Pt NC nanocrystallites, (iii) creation of hollow interiors through Ostwald ripening mechanism, resulting in hollow nanostructures, and (iv) oriented attachment of Pt NCs along <100> direction and transformation of unusual hollow truncated octahedra to perfection of hollow nanotetrahedra. It is noted that, the Kirkendall effect is based on difference between diffusion rates of two elements (D_A_≠D_B_), yielding a layer of shell materials (AB) upon thermal treatment. Because hollow nanocrystals are especially synthesized without thermal treatment and also there is only a single phase (i.e., Pt NCs), the Kirkendall mechanism can be ruled out unambiguously^29-30^.

Based on previous reports, the OA growth kinetics is an effective controlling factor to determine the shape and size distribution for nanoscale synthesis via surface capping. The formation of platinum crystal aggregates is attributed to utilizing Hb as capping agent in the synthesis. Replacing Hb with BSA induced an opposite behavior for Pt NCs formation with controlled morphology (Figure S7), demonstrating that the strong surface adsorption of Hb on the crystal planes of the Pt NC nanocrystallites simplifies the nanocrystal growth through OA-based growth^27, 31-32^. In this case, high surface reactivity of Pt_n_ clusters due to their small size and high conformational flexibility of hemoglobin lead to obtaining other crystal morphologies and general size-control using OA and Ostwald ripening processes based on time-dependent experimental results.

Comparing Pt NC aggregates formation at 37 ºC and 67 ºC showed that the increasing in reaction rate by rising temperature produces bigger particles with a broader size distribution, because of the effect of simultaneous nucleation and crystal growth in synthesis solution (Figure 4a-f); in the other word, the previously formed nuclei grow faster and become larger than those formed later, resulting in a broad size distribution^33^. Furthermore, the presence of the small number of hollow tetrahedrons and porous spheres implies that shape transformation was happened from hollow nanocrystals to porous microspheres for a short time. As shown in Figure 4a-f, particles with large sizes also attach to each other via OA and self-recrystallize into a larger particles with round shapes and irregular geometries, according to the relation of volume between the particles attached and cluster aggregates, which are tens to thousands times larger than a small cluster^32, 34^. It should be mentioned that there may be the number of probabilities of OA-based growth kinetic models in the system according to the Smoluchowski theory, indicating collisions and attachments among particles, i.e. the primary particles (A1+A1), primary particles and secondary particles (A1+A2), primary particles and a multilevel particle (A1+Ai), and the collision and coalescence between any of two multilevel particles (Ai +Aj) (I ≥ 2, j ≥ 2)^31-32^. From the above results, it is believed that the hemoglobin-capped Pt NCs are primary particles which act as the ‘‘active centers’’ to induce coalescence and oriented attachment with other particles^31, 35^. Moreover, a large number of multilevel particles implies the high possibility of OA among multilevel nanoparticles which lead to control the multilevel OA dominant growth stage over a relatively short time period; Besides, a large number of porous spheres and a low number of hollow structures verify that the high temperature-induced growth enhancement can limit the dissolution of particles during the OR processes which may in turn reduce the number of hollow structures^32^. As can be seen in Figure. 4d-f, the formed multilevel particles are also surrounded by the strong surface adsorbent of Hb with relative stability during coarsening; this ability is due to the strong interaction between Hb and nanoaggregate surfaces; all of which indicate that the high temperature results in a better oriented attachment among the crystallites, and a denser packing for the final crystal spheres, i.e. OA is dominant process compared to OR processes for controlling the multilevel growth stage^35^. Also, when particle coalescence takes places, removing adsorbed molecules (e.g., hydroxyls, protons, and water molecules) from the particle-particle interface may facilitate the reorientation and conversion to an oriented aggregate^35^.

Moreover, from the FESEM analysis (Figure 4d-f), it is found that desorption of surface Hb capping agent does not take place with rising temperature; this probably prevents equilibrium saturation; therefore, the OR growth is prohibited thermodynamically^31-32, 34^. As the zeta potential indicates, the strong force of electrostatic repulsion between the particles (-33.6 ± 3 mV) is also responsible for the formation of compact, porous clusters at 67 ºC^28^. Additionally, with increasing cluster size, the collision cross-section of multilevel particles grows up and their motion rate reduce, both of which help to increase the size of cluster nanoaggregates during OA multilevel growth which will finally stop^31^. According to the above-mentioned discussions, in a word, because the OA of nanoparticles plays a large role in the growth of monodisperse platinum nanocrystals and in controlling the size, shape, as well as crystallinity of nanocrystal aggregates, the OA growth factors, such as type of capping agent, temperature, and reaction time on the synthesis of nanoclusters in the solution, should be considered^32, 35^.

# Electrocatalytic performance of Pt NC aggregates.

**Pt Loading.** The composition ratios of Pt were evaluated by energy dispersive X-ray spectrometry (EDS) and inductively coupled plasma–atomic emission spectroscopy (ICP-AES) in the final products (Figure S9). ICP-AES exhibited that the actual composition ratios of Pt in the synthesized NCs were 0.21, 0.13, and 0.06% for the Pt NCs prepared at 67 ºC, 37 ºC, and 37 ºC in the presence of NaBH_4_, respectively, also indicating similar composition of Pt in crystalline nanocluster aggregates. The low amount of Pt in the final products is also confirmed by EDX analysis (Figure S9) which may be due to low Pt precursor concentration, 0.125 mM (24.2 ppm) needed for the direct synthesis of hemoglobin stabilized platinum nanoclusters. Moreover, EDX analyses on the Hb/Pt NCs gave clear platinum signals; however, iron peak related to heme group of hemoglobin did not appear, probably due to the small amount of Fe in Pt crystals^36^ (Figure S9).


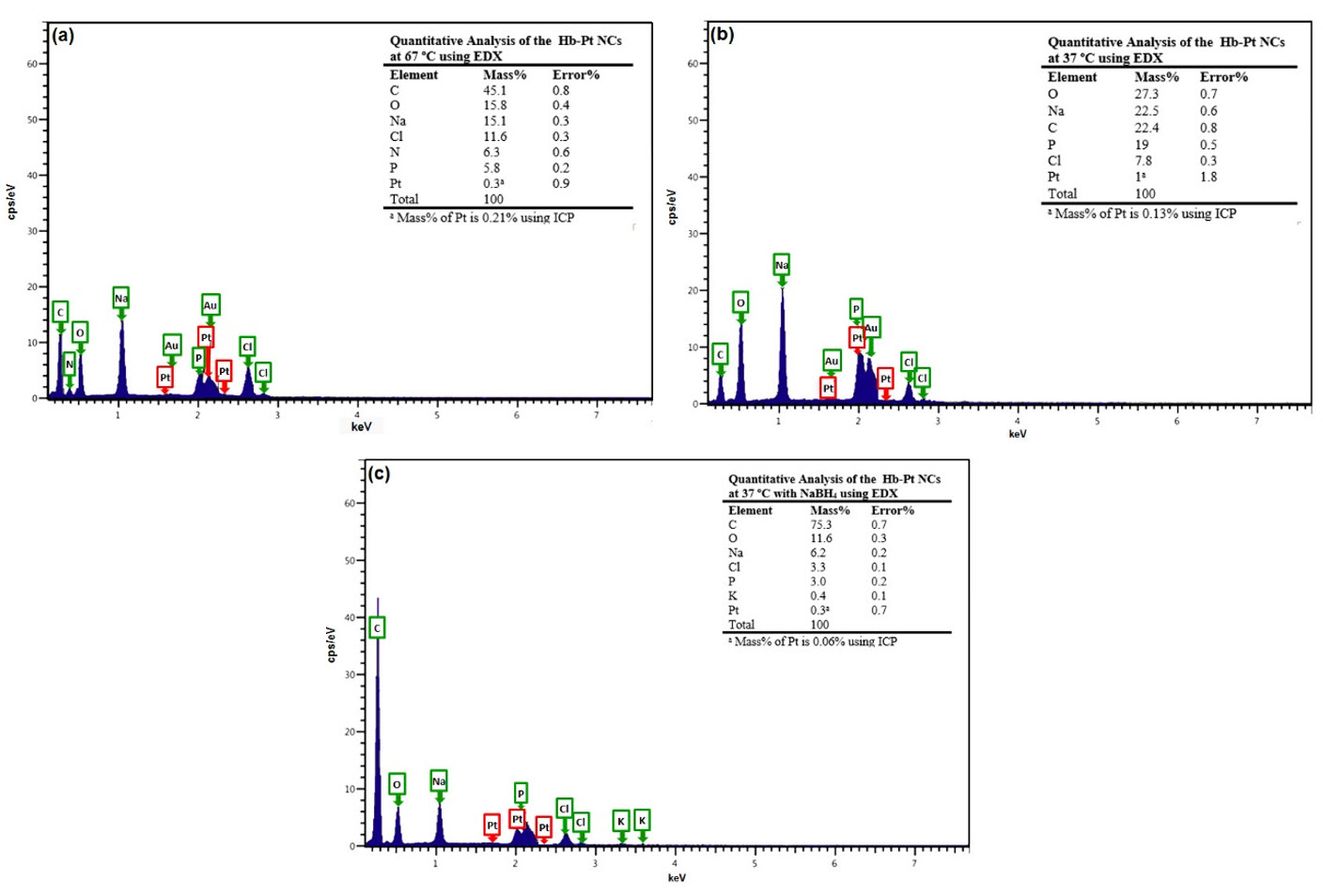


Figure S9. EDS spectra of Hb/PtNCs prepared at (a) 67ᵒC (b) 37ᵒC and (c) 37 °C by using NaBH_4_ (1cc of 40 mM). Insets show EDS and ICP analyses of as-prepared Hb/PtNCs.

Loading amount of Pt is calculated by following method:

Concentration of catalysts × loading of catalysts on electrode × wt% of catalysts

For example: 2 mg × 5 μl × 0.21/100 ÷ 1000 μl = 0.000021 mg

In respect of area, the amount of Pt = 0.000021/0.0314 = 0.00068 mg/cm^2^ = 0.68 μg/cm^2^

**Note:** Ultra-low Pt loading in this work was obtained under aggregation and hollowing processes resulting high active site and interesting morphology, thereby leads to extraordinary ORR activity. Considering the onset potential in ORR, although Pt NC aggregates showed the cathode activation overpotential caused by the sluggish proton transfer thorough protein layer, compared to that of traditional noble metal-based and some Pt-free catalysts, the present Pt NC aggregates in particular porous nanospheres and after that nanotetrahedra actually exhibit catalytic activity comparable with or even better than that of other non-Pt materials and clusters^37-40^.


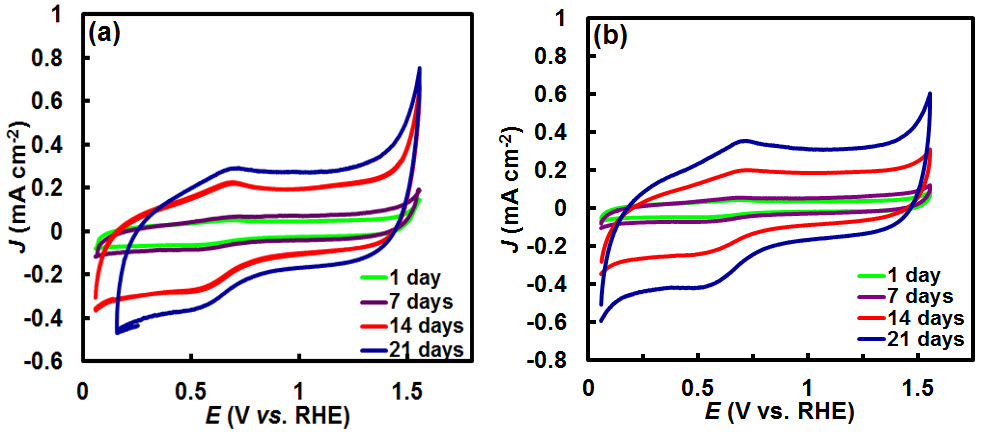


Figure S10. CVs of the Pt NCs obtained at various times in an (a) N_2_- and (b) O_2_-saturated 0.1M HClO_4_ solution with the cyclic potential sweeping between 0 and 1.5V versus reversible hydrogen electrode (RHE) at a scan rate of 100 mVs^-1^.


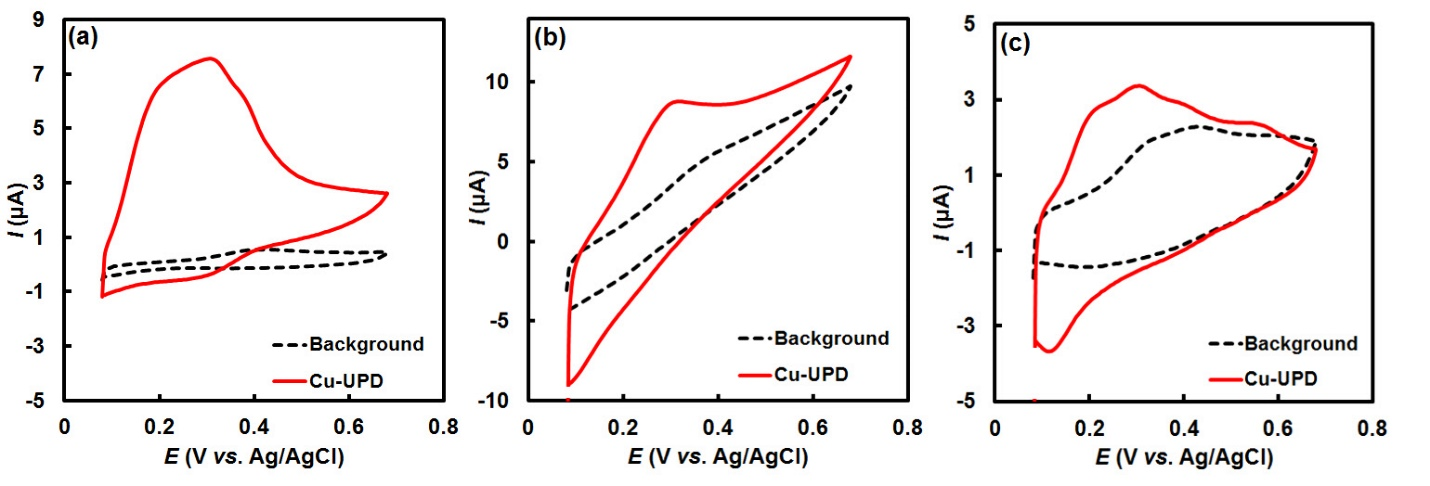


Figure S11. Cu_UPD_ stripping measurements of the PtNC hollow tetrahedra (a), PtNC porous microspheres (b), and PtNC hollow polyhedra (c) in solution of 0.5 M H_2_SO_4_ + 1 mM CuSO_4_ at 20 mV/s.


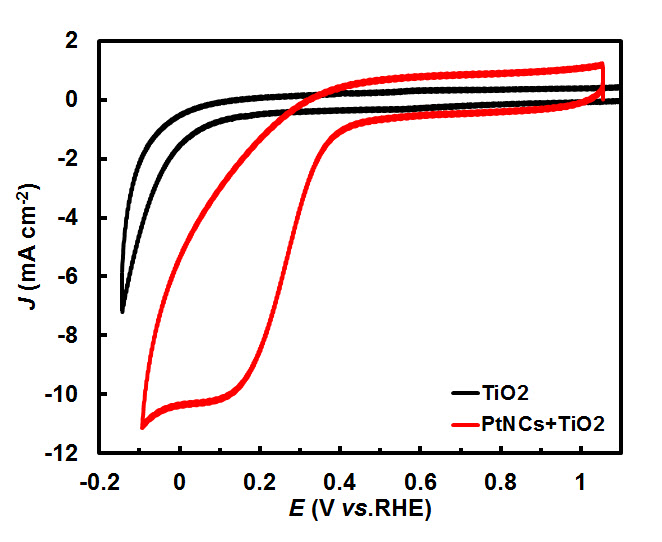


Figure S12. CVs of TiO_2_ and PtNCs + TiO_2_ in an O_2_-saturated 0.1M HClO_4_ solution with the cyclic potential sweeping between -0.2 and 1.0 V versus reversible hydrogen electrode (RHE) at a scan rate of 100 mVs^-1^.


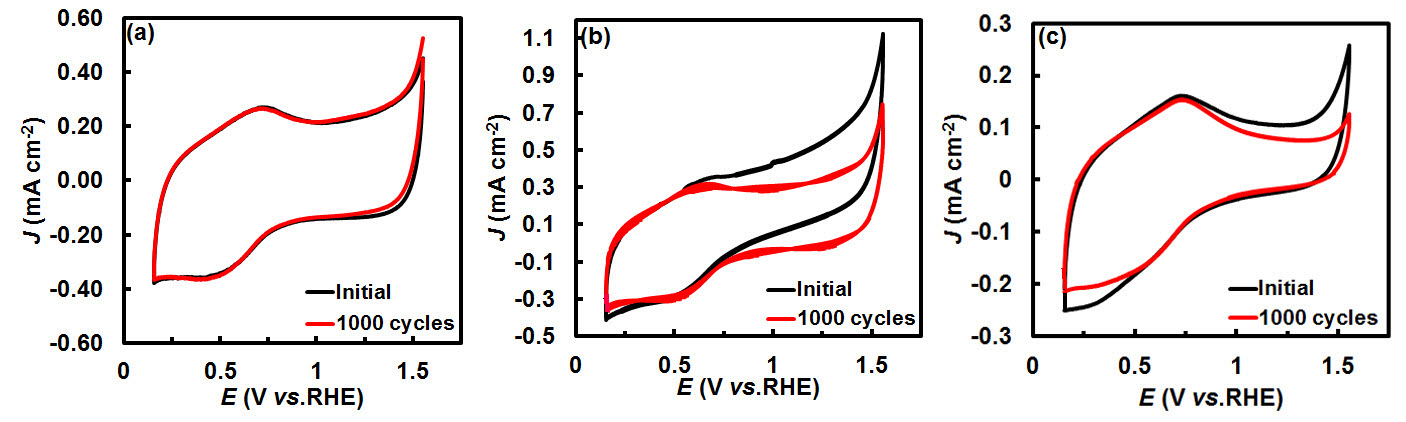


Figure S13. CVs of the PtNC porous microspheres (a), PtNC hollow tetrahedra (b), and PtNC hollow polyhedral (c) obtained before (black) and after (red) 1000 potential sweeps vs. RHE in an O_2_-saturated 0.1M HClO_4_ solution with a sweep rate of 100 mVs^-1^.

**Note:** the long-term stability of the Pt NC catalysts was performed by cyclic voltammetry in a N_2_-saturated 0.1M HClO_4_ solution between 0 and 1.4V (Data not shown). Measuring H adsorption shows no change for PHP and also increased Pt surface area for PPM and SCHT, indicating no loss of Pt surface area or/and activity, while there is a large drop of 21% in ECSA after1000 cycles for Pt/C with deposited amount of 10 times more than the prepared Pt catalysts ^41^. The decrease in stability of Pt/C during the ORR is commonly related to the corrosion of the carbon, which results in the detachment of Pt nanoparticles from the carbon support and Ostwald ripening/aggregation of Pt nanoparticles supported on carbon^42^, while from the results of the long-term stability and ADT tests, all of the PtNC aggregates showed higher stability than Pt/C. Considering the ECSA loss of Pt/C catalysts over a long period of time during ORR (between 20-60%).

# Cancer cell targeting imaging and cytotoxicity assay.


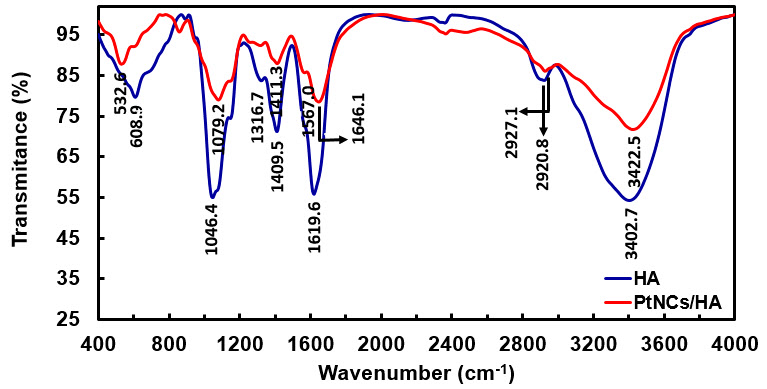


Figure S14. FTIR spectra of HA (blue) and Pt NCs/HA (red).

# Experimental section

## Materials.

All reagents used were of analytical grade and were used as received without further purification. Chloroplatinic acid hexahydrate (H_2_PtCl_6_.6H_2_O), ethanol (CH_3_CH_2_OH), sodium borohydride (NaBH_4_), perchloric acid (HClO_4_), 1-Ethyl-3-(3-dimethylaminopropyl) carbodiimide (EDC), N-hydroxysuccinimide (NHS) and Hyaluronic acid (HA) were obtained from Sigma-Aldrich (St. Louis, MO). Sodium hydroxide pellet, sodium chloride and multiwall carbon nanotubes (MWCNTs) were purchased from Merck (Darmstadt, Germany) and Pt black was purchased from Johnson-Matthey Corp. Deionized water having a resistivity of no less than 18 MΩ·cm (Milli-Q, Bedford, MA), was used as solvent and Phosphate buffered saline was used as a buffer solution throughout the experiments. All glass bottles were washed with aqua regia and rinsed with ultrapure water. Hemoglobin with high purity (> 95%) was prepared according to the method of William and Tsay and its concentration was determined by the method of Antonini and Brunori (ɛ_415nm_ = 125 mM^-1^ cm^-1^ or ɛ_541nm_ = 13.8 mM^-1^ cm^-1^ per heme), as reported before^5^. The concentrations were high enough (i.e., ≥ 100 µM) to avoid the formation of a considerable number of dimers, which should always be ≤ 5%.

## Synthesis of hemoglobin-capped Pt NCs

Platinum NCs were synthesized quite simple by using the one-step synthetic route following the procedure similar to our previously-described method for Au NCs^5^. In brief, by mixing 2.5 mL of Hb (0.11 mM) and 2.5 mL of H_2_PtCl_6_ at different molar ratios of Pt: Hb from 0.56:1 to 18.2:1 under vigorous stirring at 37 ᵒC, the blue-green luminescent Hb/PtNCs were readily prepared. Sampling of the reaction solution was done at various stirring times from 1 to 5 days for each molar ratio of Pt: Hb at which the pH was adjusted to about 12 with NaOH (1 M). Solutions were subsequently purified through centrifugation (12 000g) to remove the large platinum nanoparticles, leaving a clear Pt nanocluster solution.

In order to obtain a high concentration of Pt NCs, the key parameters such as aging time (from 0.5 h to 25 days) and reaction temperature (from 27°C to 67°C) were optimized whereby morphology control can be obtained. In this regard, the monodisperse Pt NCs with spherical and tetrahedron morphology were synthesized by incubation of the reaction solution for 10 days at 67 °C, and 27 days at 37 °C, respectively (Figure 3d-l, 4a-f). Additionally, the morphology of prepared Pt NCs was evaluated in the presence of NaBH_4_ as a strong reducing agent. (Figure 4g-l). In this way, 1 mL of freshly prepared NaBH_4_ (40 mM) was drop wise added to the Hb/Pt solution (pH ~12, 37°C) over a 15-min period under vigorous stirring. The solution color changed gradually from deep red to yellow over approximately 24 h. It has to be noted that the formation of the Pt NCs with good fluorescence intensity on the first day (24 h) is possible; however, more time results in higher quantum yield. The final products were dialyzed (24 h), freeze-dried, and stored at 4 °C until required for further characterization, as well as imaging and catalytic applications. The produced nanoclusters were diluted 100-fold with double-distilled water before analysis. No change in optical properties was observed even several months after the synthesis. Hb was also used as control under basic condition (pH ~ 12) without the use of H_2_PtCl_6_ as Pt precursor for the presented experiments.

## Synthesis of HA-Conjugated Pt NCs

10 mg of HA was dissolved in water (2 ml), then the surface carboxylic groups of HA were activated by adding EDC: NHS (1: 1 mg) and stirred at room temperature for 3 h. The Pt NCs (10 mg) were added in the mixture and incubated at room temperature for 24 h. The product was filtered (100 kD MWCO), followed by freeze-drying under high vacuum conditions.

## Structural Characterization

The samples were characterized with different analytical techniques. Fluorescence measurements were performed using a Perkin-Elmer LS-50B fluorescence spectrometer (Perkin-Elmer, UK), equipped with a xenon lamp as the excitation light source. The emission spectra were recorded in the wavelength ranges of 380-600 nm and 670-900 nm upon excitation at 360 nm and 320 nm, respectively. The scan rate was 1500 nm min^-1^. UV/Vis absorption spectra were measured with a Model Scinco UV S-2100 (Cinco, Korea), over the wavelength range of 250-800 nm. The CD spectra of pure Hb and Hb/Pt NCs in aqueous medium were recorded on a J-715 JASCO CD spectrometer (JASCO, Japan) with a cell of 1 mm path length. All spectra were collected from 190 to 250 nm and average of three scans was adopted to increase the signal to noise ratio of the CD spectra. The FTIR spectra of freeze-dried samples supported on KBr pellets were recorded using a Tensor 27 Bruker instrument (Bruker, Japan). In all cases, the data were averaged over 16 scans. The resolution of the instrument was 4 cm^−1^. The TEM and HRTEM images were recorded using an EM10C Zeiss transmission electron microscope (Zeiss, Germany) and a Philips CM30 transmission electron microscope (Philips, Netherlands) with accelerating voltages of 80 and 300 kV, respectively. TEM characterization was performed by pipetting 20 µL sample onto carbon-coated copper grids and allowing it to dry under ambient condition. Dynamic light scattering (DLS) and ζ-potential were determined using a Zetasizer Nano ZS (Malvern Instruments Ltd., U.K.) equipped with a 633 nm (He−Ne) laser. The XPS measurements were carried out on an ESCALab220I-XL spectrometer (VG, U.K.) with monochromatized Al Kα radiation at 1486.6 eV, operating at a vacuum < 10^-7^ Pa. FE-SEM images in combination with energy dispersive X-ray spectroscopy (EDX) analysis were recorded using Carl Zeiss field emission scanning electron microscope (SIGMA VP, Carl Zeiss, Germany) after drop-casting of the as-synthesized samples (20 µL) on a glass slide covered with an aluminum foil, following air-drying, and sputter-coating a gold film. The composition of platinum nanocluster samples was determined by inductively coupled plasma-atomic emission spectroscopy ICP-AES (CIROS VISION). MALDI MS analysis of Hb and Hb/Pt NCs were conducted using a Kratos Axima CFRplus (Shimadzu Biotech, Manchester, U.K.). In order to enhance ionization, matrix solution was prepared by 10 mg/mL sinapinic acid as the matrix in ACN: TFA: water (25:75:0.1); after that 2μL of the as-synthesized Hb/Pt NCs (5 mg/mL) was mixed with 40 μL of matrix solution. Finally, 2.5 µL of the resulting mixture was spotted, air-dried, and irradiated with a pulsed 337 nm nitrogen laser to form ions. Mass spectra were collected in positive-ion mode and were averaged over 5 seconds (100 laser shots).

## Electrochemical Characterization

Electrochemical measurements were carried out with the VSP-300 multichannel Potentiostat/Galvanostat/EIS (Bio-Logic Science Instruments, France)**.** A typical three-electrode configuration was employed, consisting of a modified glassy carbon electrode as the working electrode, Ag/AgCl (3 M KCl) reference electrode and plain Pt sheet (1 cm^2^) as a counter electrode in 0.1 M HClO_4_. All electrochemical data were recorded at room temperature, and all the potentials are expressed with respect to the reversible hydrogen electrode (RHE). The glassy carbon electrode (GCE) was modified with MWCNTs functionalized with carboxylic acid groups (MWCNT-COOH) for the immobilization of Pt NCs on the surface electrode whereby the durability and reproducibility of Pt NCs as catalyst can be increased. Purification and preparation of MWCNTs functionalized with carboxylic acid groups was carried out using nitric acid according to the Staudenmaier method. In brief, to 60% nitric acid (50.0 ml), the MWCNTs (5.0 mg) were added and sonicated for 6 h at 40 ºC using a bath-type sonicator (BRANSON 5510, Germany). The mixture was then filtered and washed with double-distilled water several times until equilibrium pH of the elution solution is at least above 6.0; after that, obtained solid (MWCNT-COOH) was dried under vacuum and used for modification of the GC electrode which is as follows: 1 mg of functionalized carbon nanotube with COOH was dispersed by sonication in 1 ml DMF and a volume of 10 μL of the suspension transferred onto the polished glassy carbon electrode. After evaporation of the DMF, the electrode was covered with EDC/NHS linker solution (20 mM) and maintained for 3 h in refrigerator. After washing with water, 5μl of the dispersed sample solutions (2 mg/ml) were dropped onto a GC electrode (2 mm diameter, 0.0314 cm^2^ geometrical surface area), followed by drying at room temperature Pt loading was calculated based on ICP-AES. On completion, the electrode was washed water to remove residual organic materials and stabilizing agents. Before each measurement the catalyst suspension was ultrasonicated for 3min to obtain homogeneous dispersion. Similarly, the commercial Pt/C (20 wt. % Pt, Johnson Matthey) was also dispersed in ethanol to form a catalyst ink (0.10 mg_Pt_ mL^−1^) under ultrasound. CVs were recorded by scanning the electrode potential from -0.8 to 1.5V and 0.0 to 1.5V, respectively, versus RHE at a scan rate of 100 mVs^-1^. CV measurements were carried out in an O_2_- and N_2_-saturated 0.1M HClO_4_ solution at room temperature. The oxygen reduction reaction (ORR) was measured using the catalyst-loaded GC rotating-disk electrodes (RDEs) in an O_2_-saturated 0.1M HClO_4_ solution at various rotation rates from 200 to 2500 rpm controlled through NOVA 1.9 software (Metrohm *µ*AutolabII, Japan). Before obtaining the ORR polarization curves (LSV), the cell was purged for 5 minutes with O_2_ and then linear sweep voltammograms were recorded by scanning the disk potential from -0.6 to 0.2V versus RHE at a scan rate of 10 mVs^-1^. The durability of catalysts was investigated by applying continues cyclic voltammetry to the working electrode for ca. 8 h (1000 cycles) via the conditions mentioned above.

To characterize the intrinsic ORR activities of the three catalysts, kinetic parameters were evaluated at different electrode potentials and compared in Table 1 by using the Koutecky–Levich equations [Eqs. (1)-(3)]^37^:

$$\frac{1}{J}=\frac{1}{J_{L}}+\frac{1}{J_{K}}=\frac{1}{B\omega^{1/2}}+\frac{1}{J_{K}}$$

$$B=n FC_{O}D_{O}^{2/3} \nu^{{-1}/6}$$

$$J_{K}=n F k C_{O}$$

Where J is the experimental current density, J_K_ and J_L_ are the kinetic- and diffusion-limited current densities, respectively, ω is the angular frequency (rad s^-1^), n is the number of electrons transferred in ORR, F is the Faraday constant (96485 C mol^-1^), C_O_ is the concentration of dissolved O_2_ in the electrolyte solution (1.26 × 10^-3^ mol L^-1^), D_O_ is the diffusion coefficient of O_2_ in 0.1M HClO_4_ solution (1.93 × 10^-5^ cm^2^ s^-1^), ν is the kinematic viscosity of the electrolyte solution (0.01 cm^2^ S^-1^ ), and k is the rate constant of electron transfer.

The electrochemically active surface area (ECSA) can be calculated using the following formula:

ECSA = Q_H_/(210 × Pt loading on electrode)

Where Q_H_ is the charges exchanged during the electro adsorption of H_2_ on Pt (from -0.13 to 0.2 V vs. Ag/AgCl)^3^. Since protein has a high specific surface area, the PtNCs samples showed a large double-layer charging current in the cyclic voltammogram (data has not been shown). Such large background current may increase the measurement error of ECSA^45^. Besides, Weaver et al. describe some factors that contribute to inaccurate background subtraction, such as substantial changes in the double-layer capacitance of the electrode during desorption oxidation of CO, leading to overestimation of the ECSA^46^. Green and Kucernak, however, indicated the effectiveness of using Cu_UPD_ stripping to determine electrochemical surface area of Pt electrodes^47^. So, we further tested ECSA by underpotential deposition (UPD) of Cu^48^. Typically, Nitrogen (99.999%) was bubbled through a 0.5 M H_2_SO_4_ solution for 15 minutes before the measurement and then passed over the solution during the entire procedure. A voltage of 0.01 V was applied to the working electrode for 60 seconds, in order to reduce the Platinum surface-oxide moieties. A CuSO_4_ solution was added to the working-electrode compartment to obtain a concentration of 1 mM CuSO_4_. A Cu_upd_ monolayer was created by applying 0.3 V to the working electrode for 100 seconds. The Cu_upd_ stripping voltammogram was obtained by scanning the working-electrode voltage over the range of 0.3–0.9 V at a sweep rate of 20 mV s^−1^ (Figure S11). Only the first anodic scan was used for the ECSA determination. The ECSA of the catalysts was determined under the assumption of 420 μC/cm^2^ of Cu^2+^ adsorbed.

It is noted that EDS analysis on the Pt NC aggregates shows no evidence of Pt in the catalysts due to the trace amount of Pt in the synthesis, thus ICP-OES was used to determine trace levels of Pt in Pt NC catalysts.

The accelerated degradation test (ADT) to predict the durability of the prepared Pt-catalysts were conducted by comparing the ECSA before and after 1000 CV cycling in an O_2_-saturated HClO_4_ solution between 0.6 and 1.1 V, the oxide reduction region of Pt, at 50 mV/s^41, 43-44^ (Figure. S13).

## In vitro cellular imaging and cytotoxicity

Hela (human breast cancer cell line) as HA receptor-positive and HUVEC (Human Umbilical Vein Endothelial cell line) as HA receptor-negative were used to investigate the uptake of as-obtained Pt NCs and Hb-Pt NCs/HA. Cells were seeded on a six-chamber glass slide at 1 × 10^5^ cells/well with 2 ml culture medium (PRMI medium with 10 % fetal bovine serum (FBS) and 1 wt % of penciling-streptomycin) in a humidified 5% CO2 incubator atmosphere at 37 °C incubator, after 24 h, the culture medium was abandoned and cells were treated with free Pt NCs, and Hb-Pt NCs/HA (with equivalent concentration of Pt NCs), followed by incubation at 37 °C in a humidified 5% CO_2_ atmosphere for 6 h. After that, culture media were discarded, and the cells were washed with buffer (PBS 0.01 M, pH 7.4) three times to remove any unbound Hb-Pt NCs or Hb-Pt NCs/HA before fluorescence imaging. The fluorescence images were captured by an Olympus IX-81 fluorescent microscope (Olympus Imaging System, Japan). In vitro cytotoxicity investigation of Hb-Pt NCs and Hb-Pt NCs/HA was done for both HUVEC and Hela cell lines using the 3-(4,5-dimethylthiazol-2-yl)-2,5-diphenyltetrazolium bromide (MTT) assay method: 200μL of cells, at a density of 2 × 10^4^ cells/well, were placed in each well of a 96-well plate. Then the cells were incubated for 24 h at 37 °C in a humidified 5% CO_2_-containing atmosphere. After that, the medium was removed and the cells were incubated with different concentrations of Hb-PtNCs and Hb-PtNCs/HA (with the same amount of PtNCs). We used the cells in the absence of PtNCs and Hb-PtNCs/HA as a control experiment. The cells were then incubated for an additional 24 h. Then the medium containing the Hb-PtNCs and Hb-PtNCs/HA was removed, and a total of 20 μL of a stock solution containing 15 mg of MTT in 3 mL of PBS was added and incubated for an additional 4 h. Finally, 180 μL of MTT solubilizing agents was added to the cell, and properly shaken for 15 min. The absorbance was measured at a wavelength of 570 nm. The relative cell viability was measured by comparing with the control well containing only the cell.

# References

1. Le Guével, X., Trouillet, V., Spies, C., Jung, G. & Schneider, M. Synthesis of yellow-emitting platinum nanoclusters by ligand etching. *The Journal of Physical Chemistry C* **116**, 6047-6051 (2012).

2. Chen, J., Herricks, T., Geissler, M. & Xia, Y. Single-crystal nanowires of platinum can be synthesized by controlling the reaction rate of a polyol process. *Journal of the American Chemical Society* 126, 10854-10855 (2004).

3. Goswami, N. et al. Copper quantum clusters in protein matrix: potential sensor of Pb^2+^ ion. *Analytical chemistry* **83**, 9676-9680 (2011).

4. Tanaka, S.-i. et al. Synthesis of green-emitting Pt 8 nanoclusters for biomedical imaging by pre-equilibrated Pt/PAMAM (G4-OH) and mild reduction. *Optical Materials Express* **3**, 157-165 (2013).

5. Shamsipur, M., Molaabasi, F., Shanehsaz, M. & Moosavi-Movahedi, A.A. Novel blue-emitting gold nanoclusters confined in human hemoglobin, and their use as fluorescent probes for copper (II) and histidine. *Microchimica Acta* **182**, 1131-1141 (2015).

6. Dou, X. et al. Lighting up thiolated Au@ Ag nanoclusters via aggregation-induced emission. *Nanoscale* 6, 157-161 (2014).

7. Luo, Z. et al. From aggregation-induced emission of Au (I)–thiolate complexes to ultrabright Au (0)@ Au (I)–thiolate core–shell nanoclusters. *Journal of the American Chemical Society* 134, 16662-16670 (2012).

8. Jia, X., Yang, X., Li, J., Li, D. & Wang, E. Stable Cu nanoclusters: from an aggregation-induced emission mechanism to biosensing and catalytic applications. *Chemical Communications* 50, 237-239 (2014).

9. Jia, X., Li, J. & Wang, E. Cu nanoclusters with aggregation induced emission enhancement. *Small* 9, 3873-3879 (2013).

10. Wu, Z. & Jin, R. On the ligand’s role in the fluorescence of gold nanoclusters. *Nano letters* **10**, 2568-2573 (2010).

11. Chen, P.-C., Chiang, C.-K. & Chang, H.-T. Synthesis of fluorescent BSA–Au NCs for the detection of Hg2+ ions. *Journal of nanoparticle research* **15**, 1-10 (2013).

12. Chen, Y. et al. Photoemission mechanism of water-soluble silver nanoclusters: Ligand-to-metal–metal charge transfer vs strong coupling between surface plasmon and emitters. *Journal of the American Chemical Society* **136**, 1686-1689 (2014).

13. Gao, Z., Liu, F., Hu, R., Zhao, M. & Shao, N. Lysozyme-stabilized Ag nanoclusters: synthesis of different compositions and fluorescent responses to sulfide ions with distinct modes. *RSC Advances* **6**, 66233-66241 (2016).

14. Wang, C.-W. et al. Sensitive detection of cyanide using bovine serum albumin-stabilized cerium/gold nanoclusters. *Analytical and bioanalytical chemistry* **408**, 287-294 (2016).

15. Ghosh, S., Anand, U. & Mukherjee, S. Luminescent silver nanoclusters acting as a label-free photoswitch in metal ion sensing. *Analytical chemistry* **86**, 3188-3194 (2014).

16. Anand, U., Ghosh, S. & Mukherjee, S. Toggling between blue-and red-emitting fluorescent silver nanoclusters. *J. Phys. Chem. Lett* **3**, 3605-3609 (2012).

17. Chaudhari, K., Xavier, P.L. & Pradeep, T. Understanding the evolution of luminescent gold quantum clusters in protein templates. *ACS nano* **5**, 8816-8827 (2011).

18. Le Guével, X. et al. Formation of fluorescent metal (Au, Ag) nanoclusters capped in bovine serum albumin followed by fluorescence and spectroscopy. *The Journal of Physical Chemistry C* **115**, 10955-10963 (2011).

19. Shamsipur, M., Molaabasi, F., Hosseinkhani, S. & Rahmati, F. Detection of Early Stage Apoptotic Cells Based on Label-Free Cytochrome c Assay Using Bioconjugated Metal Nanoclusters as Fluorescent Probes. *Analytical chemistry* **88**, 2188-2197 (2016).

20. Goswami, N. et al. Luminescent iron clusters in solution. *Nanoscale* **6**, 1848-1854 (2014).

21. Habeeb Muhammed, M.A. et al. Luminescent Quantum Clusters of Gold in Bulk by Albumin‐Induced Core Etching of Nanoparticles: Metal Ion Sensing, Metal‐Enhanced Luminescence, and Biolabeling. *Chemistry–A European Journal* **16**, 10103-10112 (2010).

22. Nwamba, C.O., Chilaka, F.C. & Moosavi-Movahedi, A.A. Cation modulation of hemoglobin interaction with sodium n-dodecyl sulfate (SDS). II: Calcium modulation at pH 5.0. *Cell biochemistry and biophysics* **61**, 573-584 (2011).

23. Mahato, M. et al. Hemoglobin− silver interaction and bioconjugate formation: A spectroscopic study. *The Journal of Physical Chemistry B* **114**, 7062-7070 (2010).

24. Shao, Q. et al. Electrochemical and spectroscopic studies on the conformational structure of hemoglobin assembled on gold nanoparticles. *The Journal of Physical Chemistry B* **115**, 8627-8637 (2011).

25. Volden, S., Lystvet, S.M., Halskau, Ø. & Glomm, W.R. Generally applicable procedure for in situ formation of fluorescent protein-gold nanoconstructs. *RSC Advances* **2**, 11704-11711 (2012).

26. Duchesne, P.N. & Zhang, P. Local structure of fluorescent platinum nanoclusters. *Nanoscale* **4**, 4199-4205 (2012).

27. Li, Y., Whyburn, G.P. & Huang, Y. Specific peptide regulated synthesis of ultrasmall platinum nanocrystals. *Journal of the American Chemical Society* 131, 15998-15999 (2009).

28. Viswanath, B., Patra, S., Munichandraiah, N. & Ravishankar, N. Nanoporous Pt with high surface area by reaction-limited aggregation of nanoparticles. *Langmuir* 25, 3115-3121 (2009).

29. Teo, J.J., Chang, Y. & Zeng, H.C. Fabrications of hollow nanocubes of Cu_2_O and Cu via reductive self-assembly of CuO nanocrystals. *Langmuir* 22, 7369-7377 (2006).

30. Wang, W., Dahl, M. & Yin, Y. Hollow nanocrystals through the nanoscale Kirkendall effect. *Chemistry of Materials* 25, 1179-1189 (2012).

31. Zhang, J., Huang, F. & Lin, Z. Progress of nanocrystalline growth kinetics based on oriented attachment. *Nanoscale* **2**, 18-34 (2010).

32. Zhang, J. et al. Oriented attachment kinetics for ligand capped nanocrystals: Coarsening of thiol-PbS nanoparticles. *The Journal of Physical Chemistry B* **111**, 1449-1454 (2007).

33. Kongkanand, A. et al. Achieving High-Power PEM Fuel Cell Performance with an Ultralow-Pt-Content Core–Shell Catalyst. *ACS Catalysis* 6, 1578-1583 (2016).

34. Zheng, H. et al. Observation of single colloidal platinum nanocrystal growth trajectories. *Science* **324**, 1309-1312 (2009).

35. Penn, R.L. Kinetics of oriented aggregation. *The Journal of Physical Chemistry B* 108, 12707-12712 (2004).

36. Fu, X., Wang, Y., Wu, N., Gui, L. & Tang, Y. Shape-selective preparation and properties of oxalate-stabilized Pt colloid. *Langmuir* 18, 4619-4624 (2002).

37. Chen, W. & Chen, S. Oxygen electroreduction catalyzed by gold nanoclusters: strong core size effects. *Angewandte Chemie International Edition* **48**, 4386-4389 (2009).

38. Liu, M. & Chen, W. Green synthesis of silver nanoclusters supported on carbon nanodots: enhanced photoluminescence and high catalytic activity for oxygen reduction reaction. *Nanoscale* **5**, 12558-12564 (2013).

39. Yang, X. et al. A dramatic platform for oxygen reduction reaction based on silver nanoclusters. *Chemical Communications* **50**, 234-236 (2014).

40. Wu, H. & Chen, W. Copper nitride nanocubes: size-controlled synthesis and application as cathode catalyst in alkaline fuel cells. *Journal of the American Chemical Society* **133**, 15236-15239 (2011).

41. Zhang, J., Sasaki, K., Sutter, E. & Adzic, R. Stabilization of platinum oxygen-reduction electrocatalysts using gold clusters. *Science* **315**, 220-222 (2007).

42. Kim, K.W., Kim, S.M., Choi, S., Kim, J. & Lee, I.S. Electroless Pt deposition on Mn3O4 nanoparticles via the galvanic replacement process: electrocatalytic nanocomposite with enhanced performance for oxygen reduction reaction. *ACS nano* **6**, 5122-5129 (2012).

43. Escudero-Escribano, M. et al. Tuning the activity of Pt alloy electrocatalysts by means of the lanthanide contraction. *Science* **352**, 73-76 (2016).

44. Du, L. et al. Polyelectrolyte Assisted Synthesis and Enhanced Oxygen Reduction Activity of Pt Nanocrystals with Controllable Shape and Size. *ACS applied materials & interfaces* **6**, 14043-14049 (2014).

45. I. M. Sluch, A. J. Miranda, O. Elbjeirami, M. A. Omary, L. M. Slaughter, Interplay of metallophilic interactions, π–π stacking, and ligand substituent effects in the structures and luminescence properties of neutral PtII and PdII aryl isocyanide complexes. Inorganic Chemistry **51**, 10728-10746 (2012).

46. J. Chen, T. Herricks, Y. Xia, Polyol synthesis of platinum nanostructures: control of morphology through the manipulation of reduction kinetics. Angewandte Chemie 117, 2645-2648 (2005).

47. T. Yao, S. Liu, Z. Sun, Y. Li, S. He, H. Cheng, Y. Xie, Q. Liu, Y. Jiang, Z. Wu, Probing nucleation pathways for morphological manipulation of platinum nanocrystals. Journal of the American Chemical Society **134**, 9410-9416 (2012).

48. Z. Huo, C.-k. Tsung, W. Huang, X. Zhang, P. Yang, Sub-two nanometer single crystal Au nanowires. Nano Letters **8**, 2041-2044 (2008).
